# Hybrid Injectable Sol-Gel Systems Based on Thermo-Sensitive Polyurethane Hydrogels Carrying pH-Sensitive Mesoporous Silica Nanoparticles for the Controlled and Triggered Release of Therapeutic Agents

**DOI:** 10.3389/fbioe.2020.00384

**Published:** 2020-05-19

**Authors:** Monica Boffito, Alessandro Torchio, Chiara Tonda-Turo, Rossella Laurano, Miguel Gisbert-Garzarán, Julia C. Berkmann, Claudio Cassino, Miguel Manzano, Georg N. Duda, María Vallet-Regí, Katharina Schmidt-Bleek, Gianluca Ciardelli

**Affiliations:** ^1^Department of Mechanical and Aerospace Engineering, Politecnico di Torino, Turin, Italy; ^2^Department of Surgical Sciences, Università degli Studi di Torino, Turin, Italy; ^3^Departamento de Química en Ciencias Farmacéuticas, Instituto de Investigación Sanitaria del Hospital, Universidad Complutense de Madrid, Madrid, Spain; ^4^CIBER de Bioingeniería, Biomateriales y Nanomedicina (CIBER-BBN), Madrid, Spain; ^5^Julius Wolff Institut, Charité - Universitätsmedizin Berlin, Berlin, Germany; ^6^Department of Science and Technological Innovation, Università del Piemonte Orientale, Alessandria, Italy; ^7^BIH Center for Regenerative Therapies, Charité - Universitätsmedizin Berlin, Berlin, Germany

**Keywords:** thermo-sensitive hydrogels, polyurethane, pH-sensitive mesoporous silica nanoparticles, self-immolative polymer, triggered drug release, stimuli-responsive

## Abstract

Injectable therapeutic formulations locally releasing their cargo with tunable kinetics in response to external biochemical/physical cues are gaining interest in the scientific community, with the aim to overcome the cons of traditional administration routes. In this work, we proposed an alternative solution to this challenging goal by combining thermo-sensitive hydrogels based on custom-made amphiphilic poly(ether urethane)s (PEUs) and mesoporous silica nanoparticles coated with a self-immolative polymer sensitive to acid pH (MSN-CS-SIP). By exploiting PEU chemical versatility, Boc-protected amino groups were introduced as PEU building block (PEU-Boc), which were then subjected to a deprotection reaction to expose pendant primary amines along the polymer backbone (PEU-NH_2_, 3E18 -NH_2_/g_PEU–NH2_) with the aim to accelerate system response to external acid pH environment. Then, thermo-sensitive hydrogels were designed (15% w/v) showing fast gelation in physiological conditions (approximately 5 min), while no significant changes in gelation temperature and kinetics were induced by the Boc-deprotection. Conversely, free amines in PEU-NH_2_ effectively enhanced and accelerated acid pH transfer (pH 5) through hydrogel thickness (PEU-Boc and PEU-NH_2_ gels covered approximately 42 and 52% of the pH delta between their initial pH and the pH of the surrounding buffer within 30 min incubation, respectively). MSN-CS-SIP carrying a fluorescent cargo as model drug (MSN-CS-SIP-Ru) were then encapsulated within the hydrogels with no significant effects on their thermo-sensitivity. Injectability and *in situ* gelation at 37°C were demonstrated *ex vivo* through sub-cutaneous injection in rodents. Moreover, MSN-CS-SIP-Ru-loaded gels turned out to be detectable through the skin by IVIS imaging. Cargo acid pH-triggered delivery from PEU-Boc and PEU-NH_2_ gels was finally demonstrated through drug release tests in neutral and acid pH environments (in acid pH environment approximately 2-fold higher cargo release). Additionally, acid-triggered payload release from PEU-NH_2_ gels was significantly higher compared to PEU-Boc systems at 3 and 4 days incubation. The herein designed hybrid injectable formulations could thus represent a significant step forward in the development of multi-stimuli sensitive drug carriers. Indeed, being able to adapt their behavior in response to biochemical cues from the surrounding physio-pathological environment, these formulations can effectively trigger the release of their payload according to therapeutic needs.

## Introduction

The design of injectable therapeutic formulations locally releasing their cargo with controlled and prolonged kinetics is becoming an urgent need in the biomedical field. Indeed, such an approach is expected to open a new chapter in the treatment of pathological states, with the huge potential to progressively overcome the typical drawbacks of gold standard administration approaches (e.g., need to repeatedly administer high drug dosages, undesired side effects, drug accumulation in non-target tissues and organs). Additionally, a proper engineering of newly designed injectable formulations could eventually lead to an in depth control over their properties, thus allowing the achievement of the best delivery profiles ensuring the cargo to be released within the therapeutic window in the target tissue for the required time interval. Among the potential approaches under investigation in the scientific community, the design of hydrogels for drug release applications is gaining huge interest, with an increasing number of research works on this topic published annually (a 10–15% annual increase has been registered over the last 10 years, PubMed’s database). In their general definition, hydrogels are three-dimensional cross-linked networks able to absorb a remarkable amount of water/physiological fluids from the surrounding environment and characterized by a soft consistence, which makes them similar to living soft tissues (Ahmed, 2015; Ozcelik, 2016). Among the wide variety of available hydrogels, thermo-sensitive sol-gel systems that undergo a temperature-driven gelation with increasing temperature up to the physiological value represent a promising alternative as drug delivery systems (Boffito et al., 2014). Indeed, they are easily injectable in the sol/semi-gel state and perfectly take the shape of the defect cavity prior to complete gelation. Additionally, they can be easily loaded with therapeutic agents, which are then locally released over time in a sustained and controlled way. Interestingly, in the case of thermo-sensitive hydrogels based on amphiphilic polymers, both hydrophilic and hydrophobic drugs can be easily encapsulated at high concentration by exploiting the arrangement of the polymeric chains into micelles, which are also the driving-force for system transition from the sol to the gel state (Xi et al., 2014; Boffito et al., 2016, 2019a,b; Anggelia et al., 2019). Payload release from thermo-sensitive hydrogels is usually driven by passive diffusion, swelling/erosion or the co-presence of both diffusion and swelling/erosion phenomena (Huang et al., 2019). A further tuning of payload release kinetics and mechanism can be obtained by pre-loading therapeutic agents into nano- or micro-carriers, such as mesoporous silica or polymeric particles. For instance, Geng et al. (2011) incorporated vascular endothelial growth factor (VEGF)-loaded poly(lactic-*co*-glycolic acid) particles into Pluronic F127-based hydrogels for application in bladder reconstruction. Later, a similar approach was adopted by Chen et al. (2018) that reported the incorporation of VEGF-loaded polymeric microspheres into injectable thermo-sensitive hydrogels based on a star-shaped poly(D,L-lactic-*co*-glycolic acid)-*b*-methoxy poly(ethylene glycol) (PLGA-mPEG) block copolymer, demonstrating the capability of the gels to delay VEGF release from the particles. Pontremoli et al. (2018) reported the development of hybrid sol-gel systems based on a custom-made poly(ether urethane) (PEU) containing F127 as building block and ion-doped bioactive glasses (MBGs) in the form of nanoparticles or microspheres, demonstrating that the resulting composite formulations allowed a prolonged and sustained release of copper ions over time. Later, the same authors described similar injectable formulations co-releasing copper ions and ibuprofen (Boffito et al., 2019b). Interestingly, they demonstrated that drugs could be released from hybrid formulations with an anomalous mechanism resulting from the existence of a double diffusive barrier (i.e., the hydrogel and the mesoporous framework) the drug molecules must pass through before being released in the surrounding aqueous medium. On the other hand, in the case of therapeutic ion release, different mechanisms turned out to be involved in the progressive delivery of ion species, i.e., ion exchange reactions within the MBG framework followed by diffusion through the hydrogel network.

Additional control over payload release can be reached by providing the designed systems with the capability to respond to physical and/or biochemical cues from the surrounding environment, thus making it possible to finely modulate and trigger the release of encapsulated therapeutics. For instance, reactive oxygen species (ROS)-dependent drug release was reported by Gupta et al. (2014) from thermo-sensitive hydrogels based on a ABC triblock polymer containing a ROS-sensitive poly(propylene sulfide) building block. Near-infrared (NIR) light-induced drug delivery was achieved by embedding gold nanorods into thermo-sensitive poly(N-isopropylacrylamide) (PNIPAM) hydrogels which underwent shrinkage upon local heating of the nanorods due to NIR light irradiation, resulting in the triggered release of encapsulated drugs (Jiang et al., 2019). Carbon nanotubes were also used with the same goal by Dong et al. (2017) that exploited their photo-thermal effect to trigger the release of doxorubicin from thermo-sensitive hydrogels based on a poly(ε-caprolactone)-*b*-poly(ethylene glycol)-*b*-poly(ε-caprolactone) (PCL-PEG-PCL) triblock copolymer. However, differently from the former approach, triggered release was achieved in this case by exploiting a gel-to-sol transition occurring within the hydrogels in response to local heating induced by NIR light application. Another widely explored stimulus to achieve a triggered drug release *in situ* exploits pH variations. The design of drug delivery systems able to respond to specific pH values, thus accelerating the release of their payload in well-defined conditions, could effectively represent a successful strategy in the treatment of all pathological conditions characterized by pH changes in the surrounding milieu [e.g., tumor and chronic skin wound environments are characterized by acid and alkaline pH, respectively (Kanamala et al., 2016; Bullock et al., 2019)]. The goal of triggering payload release in response to external pH is usually achieved by chemically modifying hydrogel-forming polymers. For instance, Guar gum was block copolymerized with PNIPAM by Lang et al. (2006) to provide thermo-sensitive PNIPAM-based hydrogels with additional pH-sensitivity (slower release of sinomenine hydrochloride at pH 6.8 compared to pH 1.0). More recently, N-isopropylacrylamide (NIPAM), itaconamic acid (AIM) and β-cyclodextrin (β-CD) were copolymerized to get a polymer which aqueous solutions showed responsiveness to temperature and pH provided by NIPAM and AIM, respectively, and additional capability to encapsulate drug molecules due to β-CD moieties (Rwei et al., 2016). Similar results were also published by Roy et al. (2019) that developed pH- and thermo-responsive gels by grafting and crosslinking PNIPAM and poly(methacrylic acid) on β-cyclodextrins for preferential release of metronidazole and ofloxacin at colonic pH (i.e., 7.4) instead of stomach pH (acid pH). As an alternative to this approach, pH-sensitive release of a cargo can be also achieved by blending hydrogel-forming materials with pH-sensitive polymers. For instance, Zhang et al. (2018) developed injectable hydrogels modulating doxorubicin release in response to external pH (i.e., faster release in acid pH environment) by mixing chitosan, hyaluronic acid and β-sodium glycerophosphate. Later, a similar approach was adopted by Chatterjee et al. (2019) that recently reported the design of dual-responsive (pH and temperature) hydrogels by blending F127, N,N,N-trimethyl chitosan and polyethylene glycolated hyaluronic acid. In the same year, tissue adhesive and acid pH-responsive hydrogels were designed starting from chitosan-grafted-dihydrocaffeic acid and oxidized pullulan that underwent chemical crosslinking via a Schiff base reaction upon mixing (Liang et al., 2019).

Finally, as a last approach to design pH-sensitive drug-releasing gels, composite formulations combining cargo-loaded particles within a hydrogel vehicle phase have been also described in literature. For instance, Wang et al. (2010) encapsulated molecularly imprinted pH-sensitive nanospheres loaded with dexamethasone-21 phosphate disodium into UV-crosslinked gels as potential coating of implantable biosensors. In the same year, chitosan/poly-γ-glutamic acid nanoparticles loaded with amoxicillin were incorporated into pH-sensitive alginate-based hydrogels (Chang et al., 2010). Later, triggered camptothecin (CPT) release at mild acid pH values was achieved by loading CPT-containing nanoparticles based on an acid-sensitive β-cyclodextrin derivative (i.e., acetalated-β-cyclodextrin) into supramolecular hydrogels prepared starting from graphene oxide and poly(vinyl alcohol) (Ye and Hu, 2016). Lastly, Qindeel et al. (2019) described hybrid formulations for transdermal drug delivery resulting from the encapsulation of pH-sensitive nanoparticles into Carbopol 934-based hydrogels.

**SCHEME 1:**
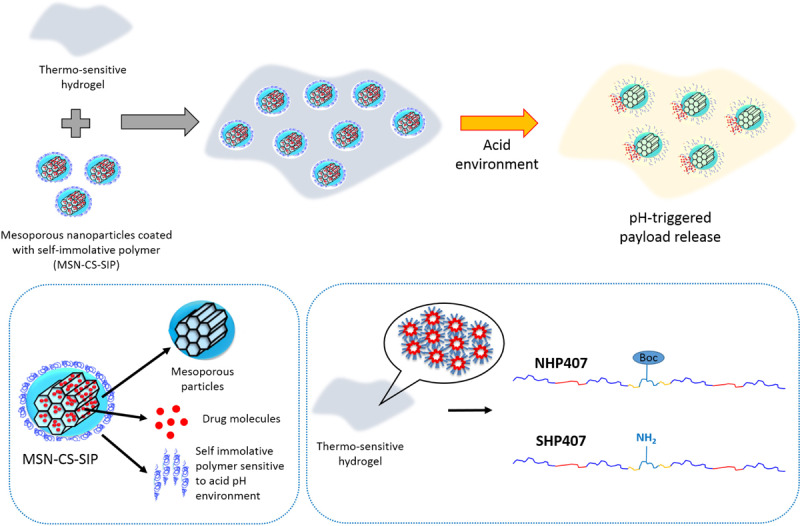
Schematic representation of the overall strategy proposed in this work to design thermo- and pH-sensitive hydrogels.

Within this constantly evolving scenario, in the present work we proposed an alternative approach to design injectable therapeutical formulations showing concurrent temperature- and pH-sensitivity, which allow easy injection and gelation under mild conditions and triggered payload release, respectively. In detail, this goal was achieved by combining the thermo-sensitivity of a custom-made amphiphilic poly(ether urethane) and the pH responsiveness of mesoporous silica nanoparticles (MSNs), which pore openings have been plugged by a self-immolative polymer (SIP) sensitive to acid pH (MSN-CS-SIP). Furthermore, to accelerate system response to pH changes, thus making acid pH transmission from the surrounding environment through hydrogel thickness faster, the wide versatility of polyurethane chemistry was exploited to introduce pendant primary amines along PEU backbone. In detail, the PEU used in this work was synthesized by chain extending Poloxamer^®^ 407 with an aliphatic non-toxic diisocyanate and an amino-acid derived diol containing Boc-protected amino-groups (NHP407). Then, amino group exposure along PEU backbone (SHP407) resulted from the optimization of the Boc deprotection procedure in terms of chloroform/trifluoroacetic acid volume ratio. The successful synthesis of NHP407 and SHP407 was demonstrated by Size Exclusion Chromatography (SEC), Fourier Transformed Infrared (FTIR) Spectroscopy, Proton Nuclear Magnetic Resonance (^1^H NMR) Spectroscopy and Orange II Sodium Salt colorimetric assay. Then, thermo-sensitive hydrogels were designed by solubilizing the PEUs in aqueous media and their gelation was qualitatively and quantitatively characterized by tube inverting and rheological tests. The capability of the hydrogels to transmit the pH of the surrounding environment through their thickness was studied by means of contact tests with buffer solutions at different pH values (pH 5 and 7.4). Finally, MSN-CS-SIP were embedded within the hydrogels and the effect of particle encapsulation on the temperature-driven sol-to-gel transition of the hybrid formulations was investigated through rheology. SIP-coated MSNs carrying a fluorescent cargo as model drug were then encapsulated within the hydrogels and its pH-triggered release was investigated by incubating the gels in neutral and acid pH media. Injectability and *in situ* dispersion and gelation were finally assessed *ex vivo* through sub-cutaneous injection in rodents. Scheme 1 summarizes the overall strategy we adopted to design the thermo- and pH-sensitive gels described in this work.

**FIGURE 1:**
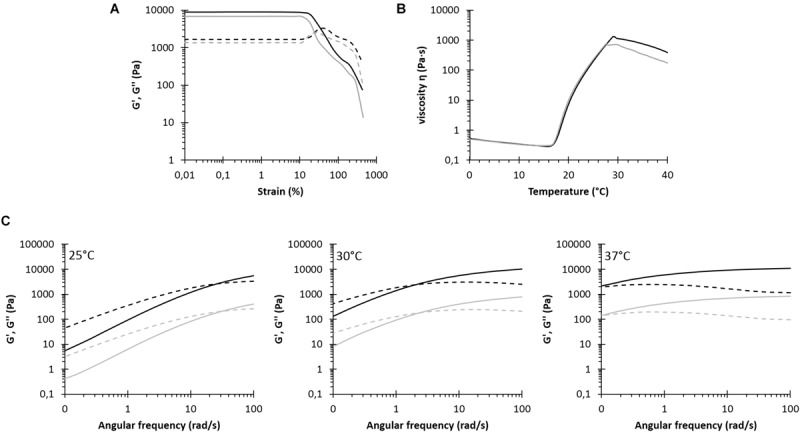
Rheological characterization of NHP407 (black) and SHP407 (gray) hydrogels. **(A)** Storage (G′, continuous line) and loss (G″, dashed line) moduli trends as a function of applied strain at 37°C. **(B)** Trend of viscosity (η) as a function of temperature within 0–40°C temperature range. **(C)** G′ and G″ trends (continuous and dashed lines, respectively) as a function of angular frequency at 25, 30, and 37°C. G′ and G″ values of SHP407 hydrogels were divided by a factor of 10.

## Materials and Methods

### Polymer Synthesis and Characterization

#### Materials

The building blocks used for PEU synthesis, i.e., Poloxamer^®^ 407 (P407, poly(ethylene oxide)-poly(propylene oxide)-poly(ethylene oxide), PEO-PPO-PEO, ${\bar{\text{M}}}_{\text{n}}$ 12600 Da, 70% w/w PEO), 1,6-hexamethylene diisocyanate (HDI) and N-Boc serinol were purchased from Sigma Aldrich, Italy. Before use, they were treated to remove residual moisture and stabilizers. Briefly, P407 was dried under reduced pressure at 100°C for 8 h and then cooled down to 30°C under vacuum, N-Boc serinol was dried at room temperature (RT) under vacuum in a dessicator, HDI was distilled under reduced pressure and stored in a dessicator until use. Butynorate was purchased from Sigma Aldrich, Italy and used as received. All solvents were purchased from Carlo Erba Reagents, Italy in the analytical grade and used as received with the exception of 1,2-dichloroethane (DCE) which was anhydrified over activated molecular sieves (3 Å, Sigma Aldrich, Italy, activation at 120°C, atmospheric pressure, overnight) at RT and under nitrogen flow for 8 h. All required glassware for PEU synthesis was dried overnight at 120°C.

#### Poly(ether urethane) Synthesis

The poly(ether urethane) used in this work was synthesized according to Boffito et al. (2016). Briefly, the synthesis was carried out in solution (i.e., anhydrous DCE) under nitrogen flow through a pre-polymerization approach. Initially, P407 was solubilized in anhydrous DCE (20% w/v) and equilibrated at 80°C. Then, HDI (2:1 molar ratio with respect to P407) and butynorate (0.1% w/w with respect to P407) were added to the P407 solution and the prepolymerization step started. After 150 min, the temperature of the reaction mixture was lowered to 60°C, N-Boc serinol was added (3% w/v in anhydrous DCE) and the chain extension reaction was carried on for 90 min. Finally, upon temperature decrease to RT and passivation of residual isocyanate groups with MeOH, the synthesized PEU was collected by precipitation in excess petroleum ether (4:1 volume ratio with respect to DCE). PEU was then purified by precipitating its solution (30% w/v in DCE) in a mixture of diethyl ether and MeOH (98:2 v/v, 5:1 volume ratio with respect to DCE). The polymer was finally collected by centrifugation (Hettich, MIKRO 220R, 6000 rpm, 0°C, 20 min), dried overnight under the fume hood and stored at 4°C under nitrogen until use.

Hereafter, the as-synthesized PEU will be referred to with the acronym NHP407, where N, H and P407 identify the chain extender, the diisocyanate and the macrodiol used for its synthesis, respectively.

#### Exposure of Free Amines Along Poly(ether urethane) Backbone

The selection of N-Boc serinol as PEU building block allowed the exposure of free amines along its backbone through a Boc-deprotection reaction. According to the protocols usually adopted in peptide synthesis reactions to deprotect Boc-protected amines of amino acids, the deprotection reaction was carried out in acid conditions (Blondelle and Houghten, 1993). Briefly, 10 g of NHP407 were solubilized in 225 ml of chloroform (CHCl_3_) at RT for 120 min (under stirring at 250 rpm) and then 25 ml of trifluoroacetic acid (TFA) were added to the solution and the mixture (overall 4% w/v polymer concentration, CHCl_3_/TFA 90/10 volume ratio) was left to react for 60 min at RT. At the end of the deprotection reaction, solvents were evaporated under vacuum using a rotary evaporator (Buchi Rotavapor Labortechnik AG) and the collected polymer was washed twice using chloroform (10% w/v) to completely evaporate TFA residues. Finally, the polymer was solubilized in distilled water (5% w/v) at 4°C overnight and dialyzed (cellulose membrane cut-off 10–12 kDa, Sigma Aldrich, Italy) against distilled water for 2 days (water refresh three times/day) to completely wash out Boc groups and residual CHCl_3_ and TFA molecules. Deprotected polymer was then freeze dried (Martin Christ ALPHA 2–4 LSC) and stored under vacuum at 4°C until use. This deprotection protocol resulted from an optimization process, which is thoroughly described in Supplementary Material.

Hereafter, the collected polymer after the Boc-deprotection reaction will be referred to with the acronym SHP407.

#### Attenuated Total Reflectance Fourier Transform Infrared Spectroscopy

Attenuated Total Reflectance Fourier Transform Infrared (ATR-FTIR) spectroscopic analyses were performed to (i) assess the success of NHP407 synthesis, and (ii) verify the absence of degradation and CHCl_3_/TFA residues in SHP407. ATR-FTIR spectra resulted from 16 scans registered at RT within the spectral range 6000–400 cm^–1^ (resolution 4 cm^–1^) using a Perkin Elmer Spectrum 100 instrument equipped with an ATR accessory with diamond crystal (UART KRS5). Spectra analysis and peak identification were conducted using the Perkin Elmer Spectrum software.

#### Size Exclusion Chromatography

Number Average and Weight Average Molecular Weights (${\bar{\text{M}}}_{\text{n}}$ and ${\bar{\text{M}}}_{\text{w}}$, respectively) and Polydispersity Index (D) of NHP407 and SHP407 samples were estimated using an Agilent Technologies 1200 Series (USA) instrument equipped with a Refractive Index detector (RID) and two Waters Styragel columns (HR2 and HR4). Analyses were conducted using tetrahydrofuran (THF, inhibitor-free, CHROMASOLV^®^ Plus, for HPLC, ≥99.9%, Sigma Aldrich, Italy) as eluent at 35°C and 0.4 ml/min flow rate. SEC samples were prepared by filtering a polymer solution (2 mg/ml in THF) through a 0.45 μm syringe filter (Whatman). Registered RID signals as a function of elution time were then analyzed using Excel software (Microsoft Corporation) to estimate ${\bar{\text{M}}}_{\text{n}}$, ${\bar{\text{M}}}_{\text{w}}$, and D relative to a calibration curve based on polystyrene standards (${\bar{\text{M}}}_{\text{n}}$ within the range 740–180,000 Da).

#### Proton Nuclear Magnetic Resonance Spectroscopy

Proton Nuclear Magnetic Resonance spectra of NHP407 and SHP407 samples were recorded in anhydrous deuterated dimethyl sulfoxide (DMSO-d6, 99.8% D with 0.03% TMS, Sigma Aldrich, Italy) by means of an Avance III Bruker spectrometer equipped with a 11.74 T superconducting magnet (500 MHz ^1^H Larmor frequency), a Bruker BBFO direct probe and a Bruker BVT 3000 unit for temperature control. The spectra were registered at 25°C and resulted from 12 scans, with 10 s relaxation time. ^1^H NMR spectra were referenced to TMS signal at 0 ppm.

#### Quantification of Exposed Amines Through Orange II Sodium Salt Colorimetric Assay

Primary amines exposed along polymer backbone upon Boc-removal were quantified according to the method recently published by Laurano et al. (2020) that adapted to water-soluble polymers the protocol usually used to quantify amino groups exposed on polymer surfaces (Noel et al., 2011). Briefly, the polymer was dissolved (0.04% w/v) in an Orange II Sodium Salt (Sigma Aldrich, Italy) aqueous solution (0.175 mg/ml in double distilled water, ddH_2_O) previously adjusted at pH 3 and then the resulting solutions were incubated for 18 h at RT, in the dark. Samples were then dialyzed against ddH_2_O (Sigma Aldrich, Italy, cellulose membrane cut-off 10–12 kDa) to wash out uncoupled dye molecules and freeze dried (Martin Christ ALPHA 2–4 LSC). Lastly, dye molecules were desorbed from lyophilized samples by solubilizing them in ddH_2_O previously adjusted at pH 12 (1% w/v) for 2 h at RT, in the dark. Finally, after centrifugation (15°C, 6000 rpm, 10 min), the samples were analyzed using an UV–Vis spectrophotometer (PerkinElmer, Lambda 25) within the spectral range from 700 to 400 nm, being Orange characteristic peak at 485 nm. Exposed amines were then quantified referring to a calibration curve based on Orange-containing standards (1.75–29.2 μg/ml concentration) prepared in ddH_2_O at pH 12.

### Hydrogel Design and Characterization

#### Hydrogel Preparation Protocol

In order to obtain thermo-sensitive sol-gel systems, NHP407 and SHP407 were dissolved at a previously optimized concentration of 15% w/v (Boffito et al., 2016). Polymer solubilization was carried out at 5°C overnight using a mix of physiological solution (0.9% NaCl), phosphate buffer at pH 8 and phosphate buffered saline (PBS, pH 7.4) at 80/10/10 volume ratio. This mixture of buffered and not-buffered aqueous solutions was selected to make the developed sol-gel systems able to more easily transport acid pH from the surrounding environment (pH 5) to their core, while forcing initial hydrogel pH toward a neutral value. In order to allow comparison among the samples and avoid results variance due to sample geometry and volume, all hydrogels (1 ml) were prepared in Bijou sample containers (Savatec, Italy, polystyrene vials with 17 mm inner diameter).

#### Tube Inverting Test

Hydrogel gelation potential was first qualitatively assessed by tube inverting test, which was performed in temperature ramp mode and in isothermal conditions at 37°C to estimate Lower Critical Gelation Temperature (LCGT) values and gelation time in physiological conditions, respectively. Briefly, LCGT values were estimated by progressively increasing hydrogel temperature from 4 to 70°C at 1°C/step, each step consisting in sample equilibration at the predefined temperature for 5 min followed by vial inversion for 30 s. At each step, “sol,” “semi-gel,” and “gel” states were defined by observing the presence of flow during the 30 s of vial inversion. Gelation time in physiological conditions was estimated by keeping the temperature constant at 37°C and inverting the vials after predefined time intervals of incubation at that temperature (i.e., from 1 to 10 min, 1 min/step). Conditions of “sol,” “semi-gel,” and “gel” were defined through vial inversion for 30 s.

#### Rheological Characterization

To better characterize hydrogel transition from the sol to the gel state, rheological characterization was performed through strain sweep, frequency sweep and temperature ramp tests. Strain sweep tests were conducted at 37°C and constant frequency (10 Hz) within the deformation range from 0.01 to 500% to characterize the designed gels in terms of their resistance to applied strain through the definition of their linear viscoelastic region (LVE, i.e., the strain range is which storage modulus -G′- is constant), the linearity limit (γ_L_, i.e., the limiting value of the LVE region) and the yield stress (YS, i.e., the value of shear stress at the maximum of the loss modulus -G″-). Then, frequency sweep tests were performed within the LVE region, at three different temperatures (i.e., 25, 30, and 37°C) and angular frequency ranging between 0.1 and 100 rad/s to characterize the progressive transition from the sol to the gel state. At each tested temperature, the angular frequency at G′/G″ crossover (ω_G__′__/G__″__crossover_) was determined using Matlab (MathWorks, R2016b version). Finally, the temperature-driven sol-to-gel transition was investigated through temperature ramp tests at constant frequency (0.1 Hz) and rate (2°C/min) within the temperature range from 0 to 40°C. Gelation onset temperature (T_onset_) was then identified at the minimum of viscosity. All tests were performed using a stress-controlled MCR302 Anton Paar rheometer equipped with a Peltier system for temperature control and a 50 mm parallel plate geometry. Before each analysis, the sample was poured on instrument lower plate at 0°C, heated at the test temperature, left to equilibrate for 10 min and finally analyzed. In the case of temperature ramp tests, equilibration was performed at 0°C.

#### Evaluation of Hydrogel pH Transfer Potential

Hydrogels based on NHP407 and SHP407 were qualitatively and quantitatively characterized in terms of their ability to transfer pH variation from the surrounding environment through their thickness. To quantitatively characterize hydrogel pH variation upon contact with environments at different pH values, samples were prepared as previously described and their initial pH was measured (Martini Instruments, Mi150, United States) in the sol state while keeping them in a water bath at 5°C. After complete gelation at 37°C (Memmert IF75, Schwabach, Germany) for 10 min, 1 ml of a buffer solution (phosphate buffered saline -PBS- pH 7.4 or acetate buffer at pH 5 (0.1 M), previously equilibrated at 37°C) was added to each sample. At predefined time intervals (15, 30, 45 min, 1 and 24 h), the residual buffer was withdrawn, the samples were incubated at 5°C to allow their sol-to-gel transition and finally hydrogel pH was measured. At each time point, hydrogel pH change was calculated according to Eq. 1.

(1) $$HydrogelpHchange\left(\%\right)=\frac{p\,H_{t\,o}-p\,H_{t\,i}}{p\,H_{t\,o}-p\,H_{b\,u\,f\,f\,e\,r}}\cdot 100$$

where *pH*_*to*_ and *pH*_*ti*_ are the measured pH values of hydrogels before (at *t*_*o*_) and after incubation in the presence of a buffer for *t*_*i*_ minutes, and *pH*_*buffer*_ is the pH of the buffer (7.4 or 5) put in contact with the hydrogels.

Qualitative evaluation of the pH gradient moving through gel thickness was performed adding pH indicators to the hydrogels (phenol red, 0.1 mg/ml, for neutral/basic pH, and bromocresol purple, 0.1 mg/ml, for acid pH, Sigma Aldrich, Italy), which change their color in response to pH changes. Upon gelation at 37°C for 10 min, the prepared hydrogels containing pH indicators were incubated in the presence of acetate buffer at pH 5 or PBS according to the previously described protocol and then photographed at predefined time intervals.

#### Hydrogel Swelling and Stability in Aqueous Environment

The capability of the developed hydrogels to absorb fluids from the surrounding environment and undergo swelling/erosion phenomena was evaluated according to a recently published protocol (Boffito et al., 2019b). In detail, hydrogels were prepared as previously described and weighed (*w*_*i*_). Upon gelation at 37°C for 10 min, 1 ml of buffer (PBS or acetate buffer at pH 5, equilibrated at 37°C) was added upon each gel and samples were again incubated at 37°C to simulate physiological conditions. Complete buffer refresh was performed every other day. At predefined time points (6 h, 1, 3, 7, and 14 days), residual buffer was removed and samples were weighed in their wet and dried states upon lyophilization (*w*_*f*_ and *w*_*f_dried*_, respectively). Control samples, i.e., samples not-subjected to swelling and stability tests, were also prepared and freeze dried to evaluate hydrogel initial dried weight (*w*_*i_dried*_). Finally, the percentage of hydrogel swelling (i.e., gel mass change in wet conditions) and dissolution/degradation (i.e., gel mass change in dry conditions) was evaluated according to equations reported by Boffito et al. (2016). In addition, at each time point swelling and dissolution/degradation data were correlated through the evaluation of the swelling ratio according to the formula reported by Park et al. (2009).

#### *Ex vivo* Characterization of the Developed Hydrogels

To test injectability and gelation potential of SHP407 hydrogel within an organism, a proof of concept cadaver study was carried out. In line with the 3R principles, the mice employed in this study had to be finalized for reasons beyond the outlined experiments. No additional animals had to be sacrificed, thereby exploiting the “reduce” of the 3R principles. The study was performed in accordance with the German Animal Welfare Act and was approved by the local animal protection authorities (LaGeSo; permit number: G 0293/17). Mice (*n* = 2) were finalized in deep anesthesia achieved by intraperitoneal (i.p.) injection of medetomidine and ketamine [medetomidine 1 mg/kg BW (Cepetor^®^, CP-Pharma, Germany) and ketamin 75 mg/kg BW (Inresa Arzneimittel, Germany)] followed by cervical dislocation directly prior to the injection of the SHP407 hydrogel (15% w/v). The rodents’ physiological body temperature was maintained by a heating plate set to 37°C and exposure to red light. The external temperature of the immediate environment of the cadaver was controlled using a thermometer. SHP407 hydrogel was stained with food coloring for better visualization, kept on ice until usage, and 200 μl were injected subcutaneously (s.c.) into the neck region using a 18G needle. 5 min post injection, the skin pocket containing the injected hydrogel was opened and gel distribution and gelation were inspected visually and haptically. Additionally, an external fixator (RISytems, Switzerland) was mounted on the right femur, and a 0.7 mm osteotomy gap was created using a giggly saw. Via the 18G needle, the blue-colored gel was applied into the fracture gap in order to test the gel distribution in a wound cavity.

### Particle Synthesis and Characterization

#### Materials

The following compounds were purchased from Sigma Aldrich Inc.: Tetraethyl orthosilicate (TEOS); Ammonium nitrate; Cetyltrimethylammonium bromide (CTAB); 4-Aminobenzyl alcohol (ABA); Phenyl chloroformate; N,N-Diisopropyl- ethylamine (DIPEA); Dibutyltin dilaurate (DBTDL); *tert*-butanol (*t*BuOH); Tris(2,2′-bipyridyl)dichlororuthenium(II) hexahydrate (Ru); (3-chloropropyl)triethoxysilane; Di-*tert*-butyl dicarbonate (BOC_2_O); Dimethyl sulfoxide (DMSO); N,N-Dimethylformamide (DMF); Tetrahydrofuran (THF); Dichloromethane (DCM). The rest of the chemicals (ethanol, heptane, etc.) were of the best quality and employed as received.

#### Synthesis of Mesoporous Silica Nanoparticles

Mesoporous silica nanoparticles (MSNs) were synthesized following the Stöber method with some modifications (Baeza et al., 2014). Briefly, CTAB (1 g, 2.74 mmol), H_2_O (480 ml) and NaOH (3.5 ml) were added to a 1l flask. Then, the solution was heated to 80°C and TEOS (5 ml, 22.39 mmol) was added dropwise over 20 min. Once the addition was completed, the solution was heated for further 2 h at 80°C under magnetic stirring. After that, the precipitate was centrifuged and washed twice with water and once with ethanol. Afterward, the surfactant was removed to obtain empty pores. For that purpose, the particles were refluxed in 350 ml of a NH_4_NO_3_ solution (10 mg/ml) in EtOH (95%) at 75°C for 2 h and subsequently centrifuged (Sorvall LEGEND XTR Centrifuge, Thermo Scientific; 9000 rpm, 15 min, 10°C). The process was repeated two more times. Finally, the nanoparticles were centrifuged, washed twice with water and once with ethanol and stored in absolute ethanol.

#### Synthesis of Self-Immolative Polymer (SIP)

(a) Phenyl(4-(hydroxymethyl)phenyl) carbamate (**1**)

Compound **1** was synthesized following a previously reported method (Gisbert-Garzarán et al., 2017). First, 4-aminobenzyl alcohol (1 g, 8.12 mmol) was dissolved in dry DMF (15 ml). Then, dry DIPEA (1.7 ml, 9.76 mmol) was added and the solution was placed in an ice bath. After that, phenyl chloroformate (1.12 ml, 8.83 mmol) was added dropwise, the ice bath was removed after 15 min and the reaction was stirred for 4 h. Afterward, the organic phase was extracted in ethyl acetate, washed with saturated ammonium chloride solution and finally dried over sodium sulfate. The solvent was partially evaporated and the resultant solution was precipitated in cold heptane and centrifuged (Sorvall LEGEND XTR Centrifuge, Thermo Scientific; 9000 rpm, 15 min, 10°C) twice in heptane.

(b) Tert-butyl(4-(hydroxymethyl)phenyl) carbamate (**2**) First, 4-aminobenzyl alcohol (1 g, 8.12 mmol) and di-*tert*-butyl dicarbonate (1.8 g, 8.12 mmol) were dissolved in dry THF (80 ml). Then, dry DIPEA (1.4 ml, 8.12 mmol) was added and the mixture was refluxed overnight. Afterward, the solvent was removed to obtain an oil that was dissolved in a small amount of ethyl acetate and precipitated in cold heptane. The solid was filtered off and dried under vacuum.

(c) Poly(phenyl(4-(hydroxymethyl)phenyl) carbamate (**3**) Compound **3** was synthesized following a modification of a previously reported method (Sagi et al., 2008). First, compound **1** (1 g, 4.12 mmol) was dissolved in dry DMSO (1.62 ml) and the solution was heated to 85°C. Then, DBTDL (5% mol) was added and the reaction was stirred for 2 h 30 min at 85°C. After that, compound **2** (223 mg, 1 mmol) in dry DMSO (0.5 ml) was injected and the solution was heated for further 2 h. Finally, the crude reaction mixture was precipitated in cold methanol, centrifuged (Sorvall LEGEND XTR Centrifuge, Thermo Scientific; 9000 rpm, 15 min, 10°C) and washed three times with methanol.

#### Synthesis of SIP-Coated Mesoporous Silica Nanoparticles

##### Mesoporous silica nanoparticles modified with (3-chloropropyl)triethoxysilane (MSN-CS)

Vacuum-dried MSNs (175 mg) were first dispersed in dry toluene (30 ml). Then, (3-chloropropyl)triethoxysilane (100 μl, 0.04 mmol) was added and the mixture was refluxed overnight. After that, the particles were isolated by centrifugation (Sorvall LEGEND XTR Centrifuge, Thermo Scientific; 9000 rpm, 15 min, 10°C), washed with toluene and ethanol and dried under vacuum.

##### SIP-coated mesoporous silica nanoparticles (MSN-CS-SIP)

First, vacuum-dried MSN-CS (175 mg) were dispersed in dry DMSO (25 ml). Separately, compound **3** (0.33 g, 0.1 mmol) was dissolved in dry DMSO (2.5 ml). Then, dry DIPEA (25.6 μl, 0.15 mmol) was added and the solution was stirred for 2 h for alcohol activation. After that, the polymer-containing solution was added dropwise to the nanoparticle dispersion and the mixture was heated to 80°C. Afterward, a second vial containing compound **3** in dry DMSO was activated for 2 h with dry DIPEA and subsequently added to the nanoparticle solution. Finally, a third vial containing compound **3** in DMSO was activated with dry DIPEA and added to the nanoparticle solution. After that, the whole reaction mixture was stirred overnight at 80°C. Finally, SIP-coated particles (MSN-CS-SIP) were centrifuged (Sorvall LEGEND XTR Centrifuge, Thermo Scientific; 9000 rpm, 15 min, 10°C), washed with DMSO, water and ethanol and dried under vacuum. For pH-triggered release experiments, the fluorescent red dye tris(2,2′-bipyridine)dichloro ruthenium (II) (Ru) was loaded into the MSN framework before SIP grafting according to Gisbert-Garzarán et al. (2017). Briefly, MSN-CS (175 mg) were incubated in a Ru solution (10.4 mg/ml in DMSO) at room temperature and under stirring for 24 h. Then, the dispersion was heated to 80°C and subjected to the previously described protocol. Ru containing MSNs will be referred to with the acronym MSN-CS-SIP-Ru.

#### Characterization of MSNs, SIP, MSN-CS, and MSN-CS-SIP

A step-by-step approach was adopted for the characterization of the synthesized mesoporous matrices and their coating, encompassing the analysis of compounds **1–3**, MSNs, MSN-CS (see Supplementary File) and culminating with the characterization of MSN-CS-SIP samples.

MSN-CS-SIP samples were characterized by Power X-Ray Diffraction (XRD) analyses performed using a Philips X’Pert diffractometer equipped with a Cu Kα radiation (wavelength 1.5406 Å). XRD patterns were registered within the 2θ range from 0.6° to 6°, with a step size of 0.02° and 5 s counting time/step. ATR-FTIR analyses were performed using a Nicolet Nexus instrument (Thermo Fisher Scientific) equipped with a Goldengate ATR accessory. Spectra resulted from the average of 64 scans within the spectral range 4000–400 cm**^–^**^1^ at 1 cm**^–^**^1^ resolution. Nitrogen adsorption and desorption isotherms were recorded on degassed samples (approximately 50 mg kept under vacuum at 40°C for 24 h) at 77 K using a Micromeritics ASAP 2020 equipment. Surface area and pore size distribution were estimated through the Brunauer-Emmett-Teller (BET) and the Barrett-Joyner-Halenda (BJH) methods, respectively. Pore volume was defined from the amount of N_2_ adsorbed at a relative pressure of approximately 0.99. Thermogravimetric (TG) analyses were conducted using a Perkin Elmer Pyris Diamond TG/DTA instrument within the temperature range from RT to 600°C (5°C/min) to quantify the amount of organic phase present in the sample (10 mg).

### Hybrid Hydrogel Design and Characterization

#### Preparation of Hybrid PEU/MSN-CS-SIP Sol-Gel Systems

Hybrid NHP407 and SHP407 hydrogels containing MSN-CS-SIP were prepared according to Boffito et al. (2019b). Briefly, PEU-based hydrogels were initially prepared according to the protocol described in section “Hydrogel preparation protocol” at higher concentration by solubilizing the polymer in the solution portion composed of physiological solution and phosphate buffer at pH 8 (0.1 M), which represents the 90% of the total aqueous solution volume required to solubilize the material at a final concentration of 15% w/v. Then, the residual 10% of aqueous solution (i.e., PBS) was used to prepare a MSN-CS-SIP-containing dispersion. In detail, MSN-CS-SIP were first dispersed at 50 mg/ml concentration in PBS through sonication (26 W, 20 kHz, Vibracell VCX130, Sonics, United States) for 3 min in a water-ice bath to avoid evaporation and then an aliquot was added to the previously solubilized PEU samples to reach final particle and polymer concentrations of 5 mg/ml and 15% w/v, respectively. Particle addition was performed with hydrogels in the sol state (at 5°C) and samples were vortexed for 30 s to homogeneously distribute the particle within the hybrid sol-gel systems.

Hereafter, MSN-CS-SIP-containing hydrogels will be referred to with the acronyms NHP407_MSN-CS-SIP and SHP407_MSN-CS-SIP.

#### Rheological Characterization of Hybrid Sol-Gel Systems

In order to investigate the effects of particle addition to hydrogels on their gelation potential, rheological characterization of the developed hybrid sol-gel systems was performed according to the previously described protocol (see section “Rheological characterization”).

#### Payload Release Test

pH-triggered release studies were performed on hybrid hydrogels encapsulating MSN-CS-SIP particles previously loaded with the red dye Ru (MSN-CS-SIP-Ru) (hydrogel acronyms: NHP407_ MSN-CS-SIP-Ru and SHP407_MSN-CS-SIP-Ru). Tests were performed according to the protocol published by Gisbert-Garzarán et al. (2017) with slight changes to adapt it to sol-gel systems. In detail, hybrid hydrogels were loaded in the sol state into 24 well cell culture inserts (transwell, Greiner, poly(ethylene terephthalate) membrane, 0.4 μm pore size) (400 μl gel/insert) and allowed to gel at 37°C for 10 min. Then, 1 ml of buffer (PBS pH 7.4 or acetate buffer at pH 5, equilibrated at 37°C) was added to each well containing an insert and release tests were conducted in physiological like conditions (i.e., in incubator at 37°C). Release medium was collected at predefined time intervals (1, 2, 4, 5, 24, 28, 72, 96, 168, and 336 h) and completely refreshed with the same volume of fresh buffer at 37°C. Collected release media were then analyzed through a plate reader (Perkin Elmer Victor X3) at a wavelength of 450 nm. For the quantification of released dye, a calibration curve was constructed starting from Ru standards prepared in PBS or acetate buffer at pH 5 at different concentrations within the range 0–200 μg/ml.

#### *Ex vivo* Characterization of Hybrid Sol-Gel Systems

The gelation and dispersion of SHP407_MSN-CS-SIP-Ru hydrogel were studied similarly to SHP407 sol-gel system as such (section “*Ex vivo* Characterization of the Developed Hydrogels”). 200 μl of composite hydrogel (SHP407_MSN-CS-SIP-Ru), SHP407 hydrogel or MSN-CS-SIP-Ru dispersion (all kept on ice until usage) were injected s.c. into the neck region of just finalized murine cadavers (*n* = 4 mice) maintained at physiological body temperature. After 5 min, the cadavers were imaged using an *in vivo* imaging system (IVIS^®^ Lumina, Caliper LifeSciences, MA; ex/em filter: 465 nm/Cy5.5). After imaging, the injection site was opened through a skin incision and the dispersion and appearance (i.e., sol or gel state) of the different injected materials were inspected visually and haptically.

### Statistical Analysis

Statistical analysis of the collected data was performed using GraphPad Prism 5 for Windows (GraphPad Software, Inc., Version 5.03, 2009)^1^. In detail, Two-way ANOVA analyses followed by Bofferoni’s multiple comparison tests were performed on data collected from swelling, dissolution/degradation, pH variation and release tests. Statistical differences were defined according to Boffito et al. (2016). Analyses were performed in triplicate and results are reported as mean ± standard deviation.

## Results

### Chemical Characterization of NHP407 and SHP407

The as-synthesized NHP407 PEU containing P407 as building block was chemically characterized by Attenuated Total Reflectance Fourier Transform Infrared (ATR-FTIR) spectroscopy and Size Exclusion Chromatography (SEC). The effects of Boc-deprotection reaction on the integrity of PEU backbone were investigated by ATR-FTIR and SEC analyses, meanwhile the effective Boc group removal was assessed by Proton Nuclear Magnetic Resonance spectroscopy. Finally, exposed -NH_2_ groups were colorimetrically quantified according to Laurano et al. (2020).

#### Characterization of NHP407 Poly(ether urethane)

Attenuated Total Reflectance Fourier Transform Infrared spectroscopic analyses were performed on both as-synthesized NHP407 and the starting P407 macrodiol for comparison (Supplementary Figure S1). As expected, NHP407 spectrum exhibited all the characteristic absorption bands of P407 at 2877 cm**^–^**^1^ (CH_2_ stretching vibration), 1242 cm**^–^**^1^ (CH_2_ rocking vibration) and 1099 cm**^–^**^1^ (asymmetric stretching of -CH_2_-O-CH_2_- groups typical of PEO blocks in P407). The formation of urethane bonds in NHP407 induced the appearance in its ATR-FTIR spectrum of new absorption peaks at 3347 cm**^–^**^1^ (N-H stretching vibration), 1720 and 1630 cm**^–^**^1^ (stretching vibration of carbonyl groups, amide I), and 1539 cm**^–^**^1^ (concurrent bending and stretching of N-H and C-N bonds, respectively). The absence of absorption peaks within 2200 and 2300 cm**^–^**^1^ proved the complete conversion of isocyanate groups.

NHP407 exhibited ${\bar{\text{M}}}_{\text{n}}$ and D values of 44600 Da and 1.42, respectively, as assessed by SEC analyses.

#### Characterization of SHP407 Poly(ether urethane)

With the aim to assess the integrity of PEU backbone upon treatment in CHCl_3_/TFA mixture (90/10 v/v), ATR-FTIR, SEC and ^1^H NMR analyses were performed on SHP407 samples and NHP407 as control. ATR-FTIR spectrum of SHP407 was completely overlapped with that of native NHP407 (Supplementary Figure S1) and no absorption peaks ascribable to residual CHCl_3_ or TFA (e.g., C-Cl and C-F stretching vibration at 600–800 cm**^–^**^1^ and 1000–1400 cm**^–^**^1^, respectively) were detected.

Estimated ${\bar{\text{M}}}_{\text{n}}$ value slightly decreased after the Boc-deprotection reaction (${\bar{\text{M}}}_{\text{n}}$ and D of SHP407 were measured to be 40700 Da and 1.56, respectively), but this change was not significant considering the typical SEC analysis error (approximately 10%) (Trathnigg, 2000).

Supplementary Figure S2 reports the ^1^H NMR spectra of NHP407 (control) and SHP407 samples. Magnified inserts in the spectral regions between 5.65–5.73 and 7.00–7.20 highlight the characteristic bands of urea and urethane N-H groups, respectively (Qin et al., 2019). The magnified insert of NHP407 spectrum within 1.31 and 1.41 ppm shows the co-presence of resonances typical of the methylene protons of HDI-deriving block (at 1.22 and 1.37 ppm) and the methyl protons of Boc caging groups (sharp singlet at 1.37 ppm, overlapped to HDI-derived signals) (Caddeo et al., 2019). Differently, the methylene protons adjacent to the urethane bonds appeared at 2.93 ppm. Upon treatment in acid conditions, the singlet at 1.37 due to Boc protons significantly decreased, indicating that the reaction conditions allowed an almost complete Boc removal (deprotection yield > 90%).

The number of exposed primary amines in SHP407 was colorimetrically quantified through the Orange II sodium salt assay. The effective exposure of amino groups along polymer chains was indirectly proved by the darker orange color of SHP407 solutions in ddH_2_O at pH 12 compared to NHP407 control samples, which exhibited a weak orange color ascribable to physical adsorbance phenomena of dye molecules to polymer chains. Assuming that orange molecules and -NH_2_ groups electrostatically interact at a 1:1 molecular ratio and subtracting the contribution of dye adsorbance to polymer chains, the number of exposed free primary amines along SHP407 was estimated to be 3.07E18 ± 1.63E17 -NH_2_/g_SHP407_.

### Characterization of NHP407- and SHP407-Based Sol-Gel Systems

NHP407- and SHP407-based hydrogels (15% w/v), prepared by solubilizing the PEUs in aqueous medium (i.e., physiological solution/PBS/buffer at pH 8 at 80/10/10 v/v), were characterized in terms of their temperature-driven gelation, capability to change their pH in response to the external aqueous medium as well as swelling and stability in aqueous environments at different pH values. Finally, injectability, gelation and gel distribution upon subcutaneous injection were evaluated *ex vivo* in a rodent model.

#### Thermo-Sensitive Behavior of NHP407 and SHP407 Hydrogels

The gelation potential of NHP407- and SHP407-based sol-gel systems was first qualitatively evaluated by tube inverting test. LCGT values were measured to be 26 and 28°C (error ± 0.5°C) for NHP407 and SHP407 hydrogels, respectively. Tube inverting test in isothermal conditions at 37°C, instead, allowed the estimation of hydrogel gelation time in physiological conditions, which turned out to be 4 and 5 min (error ± 30 s) for NHP407 and SHP407 sol-gel systems, respectively. To obtain further insight on the sol-to-gel transition of NHP407 and SHP407 hydrogels, a thorough rheological characterization was performed by strain sweep, frequency sweep and temperature ramp tests (Figure 1). Figure 1A reports the trends of storage and loss moduli (G′ and G″) at 37°C as a function of strain in the range 0.01–500% for NHP407 and SHP407 gels. As expected for structured materials, when the strain (γ) exceeded a critical value (γ_L_, limiting strain value of the LVE), G′ started to decrease, while G″ initially increased and then decreased, representing an overshoot behavior that can be correlated to a strain hardening effect. NHP407 and SHP407 hydrogels showed a similar behavior, but the NHP407-based sol-gel system was characterized by slightly higher critical deformation

(γ_L_ of 18.6 and 11.6% for NHP407 and SHP407 gels, respectively) and mechanical strength (higher G′ values within the LVE) with respect to SHP407-based one (mean G′ value within LVE for NHP407 and SHP407 gels was 8880 and 6780 Pa, respectively). Gel Yield Stress (YS) was measured to be 1790 and 875 Pa for NHP407 and SHP407 formulations, respectively. Frequency sweep tests were conducted to study the progress of gel formation and development with increasing temperature (three different temperatures were tested, i.e., 25, 30, and 37°C) (Figure 1C). Table 1 reports the G′/G″ crossover frequencies (ω_G__′__/G__″__crossover_) and the G′/G″ delta at 100 rad/s (Δ_G__′__/G_${}_{{}^{\prime \prime }}\_{}_{100\,\mathrm{rad}/s}$) for NHP407 and SHP407 gels at each tested temperature. No significant differences were observed between NHP407 and SHP407 hydrogels that were both characterized by G′ values becoming progressively constant with temperature increase, proving the progressive gel formation. At 37°C, indeed, both NHP407 and SHP407 gels turned out to be in the gel state with ω_G__′__/G__″__crossover_ lower than 0.1 rad/s. However, the kinetics of gel formation and development slightly slowed down in SHP407-based sol-gel system with respect to NHP407-based one, as shown by the slight increase in ω_G__′__/G__″__crossover_ at 25 and 30°C observed in SHP407 formulation compared to NHP407 one. The temperature-driven gelation of NHP407 and SHP407 sol-gel systems was also investigated by rheological temperature ramp tests (Figure 1B), which highlighted that SHP407-based formulation exhibited a similar gelation process compared to NHP407-based one. Table 2 summarizes the characteristic parameters extracted from the measured temperature ramp curves: the gelation onset temperature (T_onset_), viscosity at 0°C (η_0_°_C_) and viscosity value at 25°C (η_25_°_C_). As typical of solutions, viscosity initially decreased with increasing temperature until a minimum value was reached that was followed by a sharp viscosity increase. The temperature at the minimum of viscosity (T_onset_) represents the onset of the gelation process, i.e., the temperature at which polymer chains started to aggregate into micelles. The formed micelles then tended to organize into a gel network as evidenced by the viscosity increase at higher temperatures. At a certain temperature of about 29–30°C, the viscosity of both the analyzed systems decreased with increasing temperature as a consequence of melt fracture phenomena due to gel mechanical failure induced by the application of a continuous strain rate (Boffito et al., 2016).

**TABLE 1:** Frequency values at G′/G″ crossover (ω_G__′__/G__″ crossover_) and G′/G″ delta at 100 rad/s (Δ_G__′__/G_${}_{{}^{\prime \prime }}\_{}_{100\,\mathrm{rad}/s}$) for NHP407 and SHP407 hydrogels, evaluated at 25, 30, and 37°C.

	**ω_G__′__/G__″ crossover_ (rad/s)**		**Δ_G__′__/G_${}_{{}^{\prime \prime }}\_{}_{100\,\mathrm{rad}/s}$ (Pa)**	
	**NHP407**	**SHP407**	**NHP407**	**SHP407**
25°C	27.46	32.25	2130	1410
30°C	2.05	2.51	7670	5660
37°C	<0.1	<0.1	9650	7340

**TABLE 2:** T_onset_, η_0_°_C_ and η_25_°_C_ of NHP407 and SHP407 hydrogels, estimated from rheological temperature ramp tests.

	**NHP407**	**SHP407**
η_0_°_C_ (Pa⋅s)	0.57	0.50
T_onset_ (°C)	16.03	16.36
η_25_°_C_ (Pa⋅s)	201.7	230.2

#### Investigation of Hydrogel pH Transfer Potential

**FIGURE 2:**
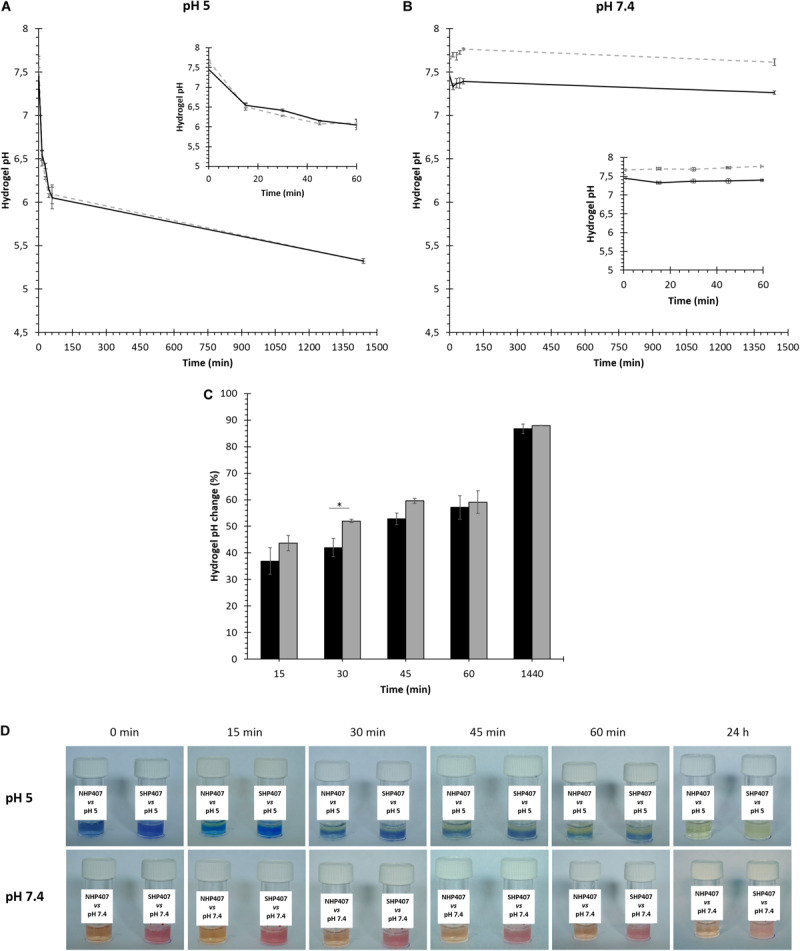
Sensitivity of NHP407- and SHP407-based gels to environmental pH (pH 5 and 7.4). **(A,B)** NHP407 (black, continuous line) and SHP407 (gray, dashed line) hydrogel pH values after incubation in buffered solutions at pH 5 or 7.4 for different time intervals. Magnified inserts evidence the trend of hydrogel pH within the first 60 min incubation in aqueous medium. **(C)** Percentage of pH change (evaluated according to Eq. 1) in NHP407- (black) and SHP407- (gray) based hydrogels upon incubation in the presence of acetate buffer at pH 5 for different time intervals. **(D)** Representative images of NHP407 and SHP407 gels containing pH indicators (phenol red for neutral/basic pH and bromocresol purple for acid pH) which change their color in response to pH changes. Gels were soaked in acetate buffer solution at pH 5 or phosphate buffered solution at pH 7.4 for different time intervals.

NHP407 and SHP407 gel ability to transfer pH changes through their thickness was tested in acid and neutral environments (i.e., pH 5 and 7.4). First, a quantitative evaluation was obtained through the measurement of hydrogel pH after incubation with buffers at different pHs (PBS, acetate buffer at pH 5) for predefined time intervals (0, 15, 30, 45 min, 1, 24 h) at 37°C (Figures 2A,B). NHP407- and SHP407-based hydrogels showed initial pH of 7.45 ± 0.10 and 7.67 ± 0.02, respectively. After 15 min contact with acetate buffer at pH 5, the pH of both hydrogels significantly decreased (0.0001 < *p* < 0.001) reaching values of 6.54 ± 0.06 and 6.50 ± 0.08 (no significant difference), respectively. However, although both the sol-gel systems reached a pH value of about 6.5, the initial pH of SHP407 hydrogels was significantly higher than that of NHP407 systems (0.01 < *p* < 0.05). Starting from 30 min incubation, hydrogel pH progressively decreased toward the value of the surrounding aqueous medium, with no significant differences between NHP407 and SHP407 hydrogels at all investigated time points (after 24 h incubation in a buffer at pH 5, NHP407 and SHP407 gels reached a pH value of 5.32 ± 0.03 and 5.32 ± 0.00, respectively) (Figure 2A). The progressive change of hydrogel pH over time was then evaluated according to Eq. 1 (Figure 2C), highlighting that pH variations in SHP407-based hydrogels showed a faster kinetics compared to NHP407-based systems up to 45 min incubation time. For instance, NHP407 and SHP407 gels covered 42.01 ± 3.42 and 52.00 ± 0.65% (0.01 < *p* < 0.05) of the pH delta between their initial pH value and the pH of the buffer (i.e., pH 5) within the first 30 min of incubation, respectively. As control condition, pH transfer tests were also conducted by soaking the gels in neutral pH environment (i.e., PBS) (Figure 2B). As expected, both NHP407 and SHP407 hydrogels did not show significant pH variations at each analyzed time point, with the exception of 24 h incubation for SHP407-based formulation (from 60 min to 24 h incubation time, SHP407 hydrogel pH significantly decreased from 7.76 ± 0.01 to 7.61 ± 0.04, 0.01 < *p* < 0.05). On the other hand, at each investigated time point the pH of SHP407 hydrogels was significantly higher than that of NHP407 sol-gel systems (0.0001 < *p* < 0.001), in accordance with its higher initial pH. After 24 h incubation in contact with PBS, NHP407 and SHP407 hydrogels exhibited pH values of 7.26 ± 0.02 and 7.61 ± 0.04, respectively. The progressive pH gradient moving through gel thickness was qualitatively evaluated observing the change in color of gels containing pH indicators and put in contact with acetate buffer at pH 5 or PBS (Figure 2D). In accordance with quantitative measurements, no changes in the color of the hydrogels were observed upon incubation in the presence of PBS, meaning that no gradient of pH change was moving through hydrogel thickness. Conversely, a progressive variation of gel color from blue to yellow was observed in gels incubated in acetate buffer at pH 5.

#### Investigation of Hydrogel Swelling and Stability in Aqueous Media

**FIGURE 3:**
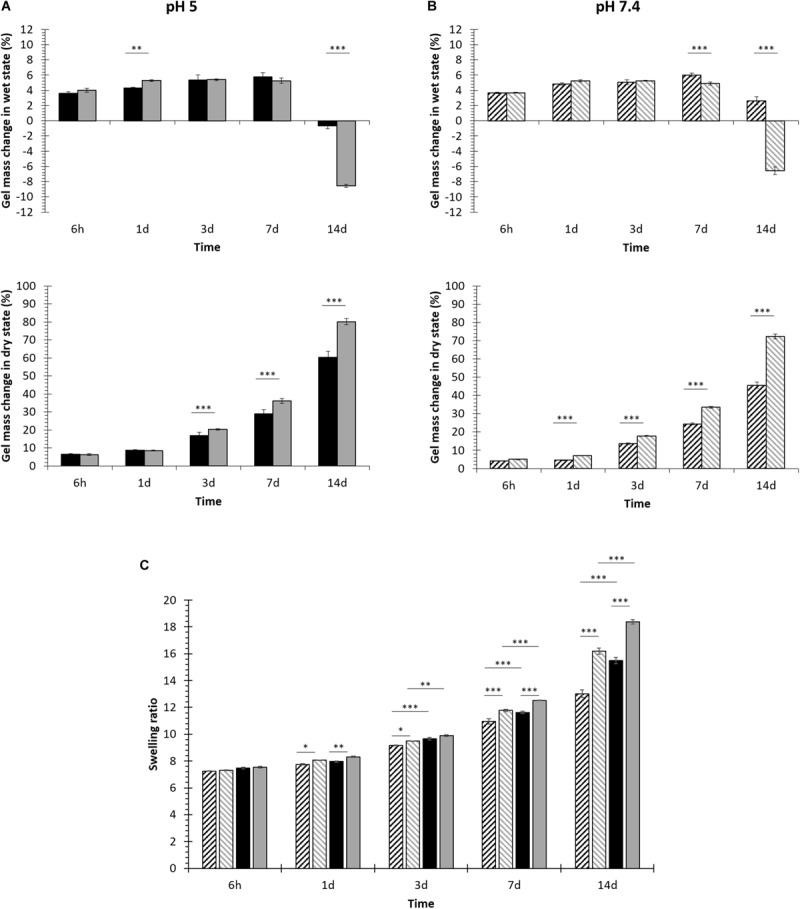
NHP407 (black) and SHP407 (gray) gel behavior in aqueous environments at different pH values. **(A)** Percentage of swelling and dissolution over time for NHP407 and SHP407 gels incubated in an aqueous medium at pH 5. **(B)** Percentage of swelling and dissolution over time for NHP407 and SHP407 gels incubated in PBS (pH 7.4). **(C)** Swelling ratio evaluated for NHP407 (black) and SHP407 (gray) gels incubated in acetate buffer at pH 5 (plain) or PBS (patterned) for different time intervals (6 h, 1, 3, 7, and 14 days).

The capability of NHP407- and SHP407-based gels to absorb fluids from the surrounding environment undergoing swelling/erosion phenomena was evaluated at 37°C by incubating the samples in contact with PBS or acetate buffer at pH 5. Gel swelling [gel mass change in wet state (%)] and dissolution/degradation [gel mass change in dry state (%)] over time are reported in Figures 3A,B, meanwhile Figure 3C reports swelling ratio data evaluated at each time point according to Park et al. (2009).

Irrespective of the surrounding environment pH, swelling prevailed over erosion/dissolution (Figures 3A,B) up to 7 days incubation for both NHP407 and SHP407 gels. However, with increasing incubation time in aqueous media, hydrogel dissolution/degradation became progressively predominant over swelling, with a consequent deswelling (i.e., a decrease in swelling percentage) which assumed negative values when erosion/dissolution completely prevailed over absorption phenomena. In the presence of a buffer at pH 5, NHP407- and SHP407-based gels showed similar swelling behavior, with significantly different swelling percentages at 1 and 14 days incubation time. In the same environment, SHP407-based gels showed significantly higher dissolution compared to NHP407-based ones starting from 3 days incubation time (0.0001 < *p* < 0.001), reaching a dissolution of 78.98 ± 0.15 and 60.61 ± 0.98%, respectively after 14 days incubation. Gels immersed in PBS showed a similar swelling trend, with SHP407 gels undergoing significant deswelling compared to NHP407 ones. Similarly to data collected from hydrogels incubated in acid pH aqueous medium, SHP407 gels exhibited higher weight loss in the dry state compared to NHP407, with significant differences (0.0001 < *p* < 0.001) from 1 day incubation on. Hence, SHP407-based sol-gel systems showed higher destabilization compared to NHP407-based ones irrespective of the environmental pH surrounding them. This consideration was also proved by the analysis of swelling ratio data (Figure 3C), that evidenced a higher swelling ratio for SHP407 gels compared to NHP407 ones at each analyzed time point, at both pH 5 and pH 7.4. On the other hand, the swelling ratio for both NHP407 and SHP407 gels was higher at pH 5 compared to pH 7.4 at each time point, with significant differences on 3, 7 and 14 days incubation.

#### *Ex vivo* Evaluation of Hydrogel Injectability and Gelation

**FIGURE 4:**
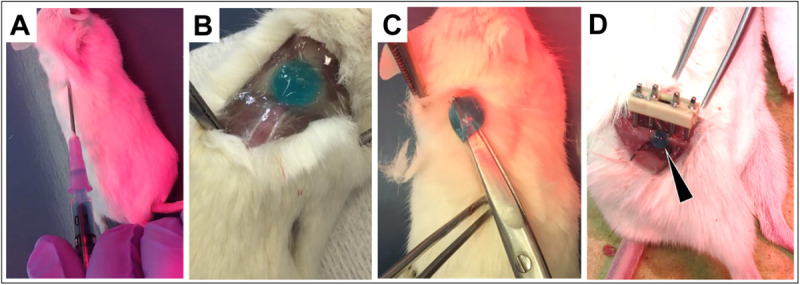
Injection and gelation of SHP407 hydrogel *in situ* into murine cadavers. **(A)** Using an 18G needle, blue-colored SHP407 hydrogel was injected s.c. into the neck region. Physiological temperature of the organism was maintained using a heating pad and red light. **(B)** Solidified SHP407 gel 5 min post s.c. injection into the neck region. **(C)** Test of solidity by lifting the SHP407 gel up. **(D)** Solid SHP407 gel (black arrow) exclusively present within the osteotomy area 5 min post injection into the fracture gap.

In order to study hydrogel injectability, dispersion and gelation *in situ* within an organism, *ex vivo* proof of concept studies were carried out working with murine cadavers. In detail, the dispersion and gelation of SHP407 hydrogel were investigated by injecting 200 μl subcutaneously (s.c.) in the neck region (Figure 4A). In Figures 4B–D, representative images of solid SHP407 gel at 5 min post injection are depicted. The blue color derives from food coloring that was previously confirmed to not affect gelation properties (data not shown). Figure 4B clearly shows a minimum dispersion of SHP407 sol-gel system as it presented as a coherent and spherical gel. Figure 4C demonstrates the solidity of SHP407 system, which allowed lifting the hydrogel using scissors. To study the dispersion and gelation of SHP407 in a more advanced application, a 0.7 mm femoral midshaft osteotomy was created in the cadaver and the hydrogel (∼15 μl) was introduced into the osteotomy gap using a 18G needle (Figure 4D). The spherical shape and the finding that the SHP407 gel remained in the gap region, without dripping onto the muscle tissue below the femur, further stresses the quick gelation and minimum dispersion *in situ*.

### Particle Characterization

**SCHEME 2:**
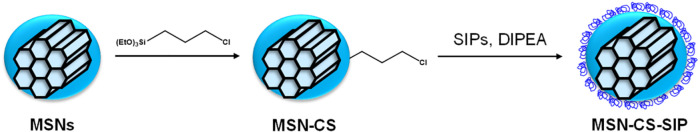
Schematic representation of the coating of MSNs with the SIP. First, the nanoparticles were surface modified with a chloro alkoxysilane (MSN-CS) and then subjected to SIP grafting, leading to SIP-coated MSNs (MSN-CS-SIP).

Mesoporous silica nanoparticles and SIP were initially synthesized and characterized separately (see Supplementary Material). In detail, MSNs were physico-chemically and morphologically characterized by XRD, ATR-FTIR spectroscopy, nitrogen adsorption and desorption analyses, TG analyses, DLS, SEM and TEM, meanwhile the SIP and its constituent blocks (i.e., compounds **1** and **2**) were characterized by ^1^H NMR spectroscopy. The functionalization of MSNs with the pH-responsive polymeric coating was accomplished following a two-step synthetic protocol: (i) MSNs functionalization with an organosilane acting as linker between the surface and the SIP (MSN-CSs) and (ii) DIPEA-mediated alcohol activation and mixing with the particles (Scheme 2). SIP-coated MSNs (MSN-CS-SIP) resulting from the grafting of SIP on MSN surface were then characterized as MSNs as such to assess the effect of the coating procedure on particle physico-chemical and morphological properties.

#### Characterization of SIP-Coated Mesoporous Silica Nanoparticles

As observed in Supplementary Figure S3A, the diffraction maxima typical of mesoporous silica nanoparticles were still observable after the grafting of the SIP on top of mesoporous silica nanoparticles. The amount of organic matter incorporated was analyzed though TG analysis (Supplementary Figure S3B). The addition of the self-immolative coating increased the weight loss up to *ca*. 15%, compared to MSNs as such. The samples were further analyzed using ATR-FTIR spectroscopy (Supplementary Figure S3C), unrevealing the presence of new functional groups. For instance, vibration bands typical of the carbamate of the self-immolative polymer (C=O stretching vibration at 1630 cm**^–^**^1^) were present in MSN-CS-SIP ATR-FTIR spectrum. N_2_ adsorption analysis (Supplementary Figure S3D) showed a reduction of the textural parameters after the SIP coating. In detail, the specific surface area decreased *ca*. 40% after SIP grafting (from *ca*. 1000 m^2^/g for MSNs to *ca*. 650 m^2^/g for MSN-CS-SIP).

### Hybrid Hydrogel Characterization

NHP407- and SHP407-based hydrogels (15% w/v) encapsulating MSN-CS-SIP (5 mg/mL) were first characterized through rheological tests. Release tests against different pHs were conducted on hydrogels encapsulating MSN-CS-SIP previously loaded with a fluorescent dye as model drug (Ru). Finally, hybrid hydrogel injectability, gelation, distribution and detectability upon subcutaneous injection were evaluated *ex vivo* in a rodent model.

#### Thermo-Sensitive Behavior of NHP407 and SHP407 Hydrogels Encapsulating MSN-CS-SIP

**FIGURE 5:**
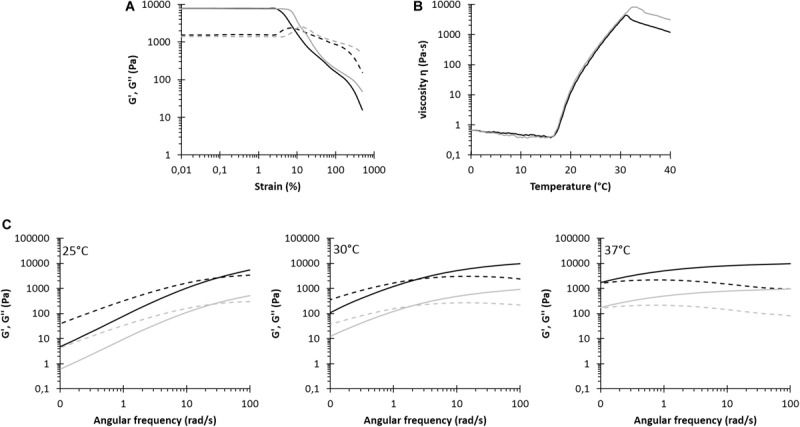
Rheological characterization of NHP407_MSN-CS-SIP (black) and SHP407_MSN-CS-SIP (gray) hydrogels. **(A)** Storage (G′, continuous line) and loss (G″, dashed line) moduli trends as a function of applied strain at 37°C. **(B)** Trend of viscosity (η) as a function of temperature within 0–40°C range. **(C)** G′ and G″ trends (continuous and dashed lines, respectively) as a function of angular frequency at 25, 30, and 37°C. G′ and G″ values of SHP407_MSN-CS-SIP hydrogels were divided by a factor of 10.

In order to assess the effects of particle addition on hybrid hydrogel gelation, rheological characterization of NHP407_MSN-CS-SIP and SHP407_MSN-CS-SIP hydrogels was conducted by strain sweep, frequency sweep and temperature ramp tests (Figure 5). Figure 5A reports the trends of storage and loss moduli at 37°C as a function of applied strain within the range 0.01–500%. Both storage and loss moduli initially showed a constant value (within the LVE region) up to the linearity limit (γ_L_) which turned out to be 2.83 and 7.25% for NHP407_MSN-CS-SIP and SHP407_MSN-CS-SIP gels, respectively. Moreover, within the linear viscoelastic region, G′ showed higher values than G″ for both NHP407_MSN-CS-SIP and SHP407_MSN-CS-SIP gels, proving that at 37°C the developed systems were in the gel state. NHP407_MSN-CS-SIP and SHP407_MSN-CS-SIP gels exhibited mean G′ values within the LVE region of 7800 and 7600 Pa, respectively, meanwhile their Yield Stress was measured to be 262 and 442 Pa. For strain value higher than γ_L_, storage modulus started to decrease, while G″ increased reaching a maximum value (2350 and 2550 Pa for NHP407_MSN-CS-SIP and SHP407_MSN-CS-SIP gels, respectively), as typical for structured networks. After this maximum value was achieved, also G″ started to decrease as a consequence of macro-cracks development throughout the sample which finally led to its complete failure, with the hydrogel behaving as a sol (i.e., G″ > G′). Hydrogel progressive transition from the sol to the gel state with increasing temperature was investigated by frequency sweep tests conducted within the angular frequency range from 0 to 100 rad/s at 25, 30, and 37°C (Figure 5C).

Table 3 reports the frequency values at G′/G″ crossover (ω_G__′__/G__″__crossover_) and the G′/G″ delta at 100 rad/s (Δ_G__′__/G_${}_{{}^{\prime \prime }}\_{}_{100\,\mathrm{rad}/s}$) for NHP407_MSN-CS-SIP and SHP407_MSN-CS-SIP evaluated at 25, 30, and 37°C. For both NHP407_MSN-CS-SIP and SHP407_MSN-CS-SIP, ω_G__′__/G__″ crossover_ value decreased with increasing temperature, as a sign of hydrogel temperature-drive gelation. On the other hand, particle encapsulation within SHP407-based systems led to lower ω_G__′__/G__″ crossover_ values compared to SHP407 hydrogels as such (at 25°C ω_G__′__/G__″ crossover_ of 32.25 and 25.96 Pa for SHP407 and SHP407_MSN-CS-SIP hydrogels, respectively) (see section “Thermo-Sensitive Behavior of NHP407 and SHP407 Hydrogels”). Conversely, MSN-CS-SIP loading within NHP407 hydrogels resulted in slightly increased ω_G__′__/G__″ crossover_ values compared to NHP407 as such (at 25°C ω_G__′__/G__″__crossover_ increased from 27.46 rad/s for NHP407 to 30.98 rad/s for NHP407_MSN-CS-SIP). Despite these differences, at 37°C both the systems appeared to be in the gel state, but complete gel development (i.e., G′ independent over frequency) was not achieved, similarly, to NHP407 and SHP407 as such.

**TABLE 3:** Frequency values at G′/G″ crossover (ω_G__′__/G__″ crossover_) and G′/G″ delta at 100 rad/s (Δ_G__′__/G_${}_{{}^{\prime \prime }}\_{}_{100\,\mathrm{rad}/s}$) for NHP407_MSN-CS-SIP and SHP407_ MSN-CS-SIP evaluated at 25, 30 and 37°C.

	**ω_G__′__/G__″__crossover_ (rad/s)**		**Δ_G__′__/G_${}_{{}^{\prime \prime }}\_{}_{100\,\mathrm{rad}/s}$ (Pa)**	
	**NHP407_**	**SHP407_**	**NHP407_**	**SHP407_**
	**MSN-CS-SIP**	**MSN-CS-SIP**	**MSN-CS-SIP**	**MSN-CS-SIP**
25°C	30.98	25.96	2060	2160
30°C	2.37	2.01	7220	6980
37°C	<0.1	<0.1	8840	8745

The trend of viscosity as a function of temperature for NHP407_MSN-CS-SIP and SHP407_MSN-CS-SIP is reported in Figure 5B, meanwhile the characteristic parameters of temperature ramp tests are summarized in Table 4. Initial viscosity slightly increased in MSN-CS-SIP-containing hybrid hydrogels compared to pure NHP407 and SHP407 sol-gel systems (η_0_°_C_ values for SHP407_MSN-CS-SIP and SHP407 systems were 0.66 and 0.50, respectively). Similarly, viscosity values of MSN-CS-SIP-containing hydrogels slightly increased compared to hydrogels as such at each tested temperature within the analyzed range; for instance, at 25°C NHP407_MSN-CS-SIP and virgin NHP407 exhibited a viscosity of 265.0 and 201.7 Pa⋅s, respectively. On the other hand, the trend of the onset temperature of gelation for SHP407-based hybrid system was opposite, with SHP407_MSN-CS-SIP hydrogel showing a slightly lower T_onset_ value compared to virgin sol-gel system. No differences in T_onset_ were observed between NHP407 and NHP407_MSN-CS-SIP.

**TABLE 4:** T_onset_, η_0_*°_C_* and η_25_*°_C_* of NHP407_MSN-CS-SIP and SHP407_MSN-CS-SIP hydrogels estimated from rheological temperature ramp tests.

	**NHP407_MSN-CS-SIP**	**SHP407_MSN-CS-SIP**
η_0_°_C_ (Pa⋅s)	0.64	0.66
T_onset_ (°C)	16.0	15.7
η_25_*°_C_* (Pa⋅s)	265.0	321.0

#### Payload Release Tests in Aqueous Environment at pH 5 or 7.4

**FIGURE 6:**
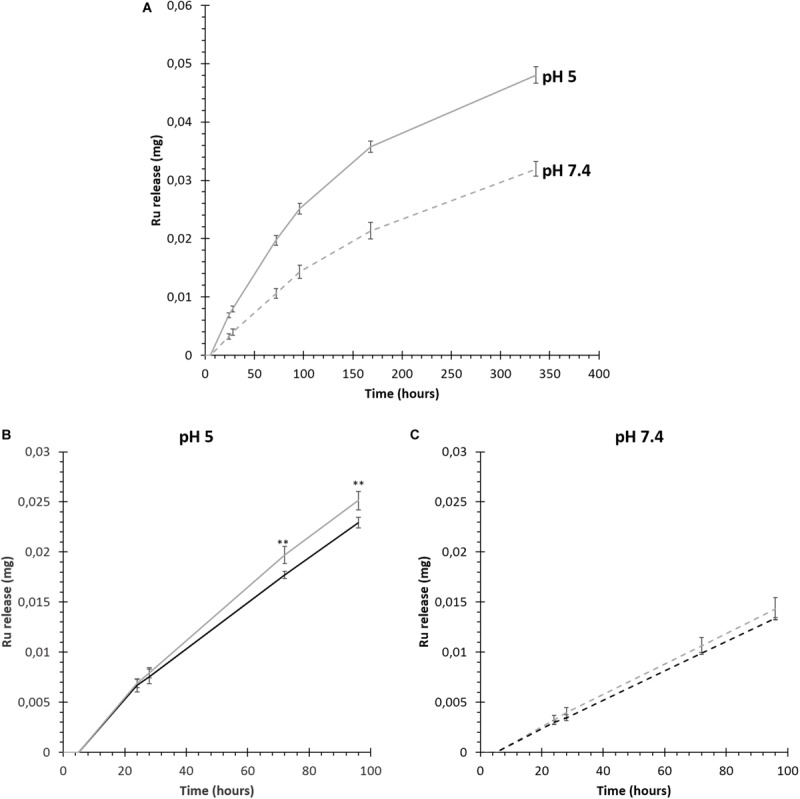
Release of Ru dye from MSN-CS-SIP-Ru-containing hydrogels (NHP407_MSN-CS-SIP-Ru in black, SHP407_MSN-CS-SIP-Ru in gray) in aqueous media at pH 5 and pH 7.4. **(A)** Ru release from SHP407_MSN-CS-SIP-Ru hydrogels incubated in acetate buffer at pH 5 (continuous line) or in phosphate buffered saline (pH 7.4) (dashed line). **(B)** Trend of Ru release in pH 5 aqueous environment from NHP407_MSN-CS-SIP-Ru (black continuous line) and SHP407_MSN-CS-SIP-Ru hydrogels (gray continuous line) within the first 4 days of observation. **(C)** Ru release in PBS (pH 7.4) from NHP407_MSN-CS-SIP-Ru (black dashed line) and SHP407_MSN-CS-SIP-Ru hydrogels (gray dashed line) within the first 4 days of observation.

The progressive release of the red dye tris(2,2′-bipyridine)dichloro ruthenium (II) (Ru) from SIP-coated MSNs embedded within NHP407 and SHP407 hydrogels (NHP407_MSN-CS-SIP-Ru and SHP407_MSN-CS-SIP-Ru) was investigated in aqueous environment at different pH values. Figure 6A reports payload release profiles over time from SHP407_MSN-CS-SIP-Ru gels incubated in pH 5 and pH 7.4 aqueous media. Similar trends were obtained also from NHP407_MSN-CS-SIP-Ru gels (data not reported). Additionally, at each analyzed time point, both NHP407_MSN-CS-SIP-Ru and SHP407_MSN-CS-SIP-Ru showed significantly higher Ru dye release in pH 5 buffer compared to PBS (0.0001 < *p* < 0.001). Furthermore, the exposure of free amines along SHP407 polymer chains induced an increased release of Ru molecules in acid pH environment from SHP407_MSN-CS-SIP-Ru compared to NHP407_MSN-CS-SIP-Ru at each analyzed time point (statistically significant differences at 72 and 96 h observation time, 0.01 < *p* < 0.05) (Figure 6B). On the other hand, at pH 7.4, Ru release from SHP407_MSN-CS-SIP-Ru was not significantly higher (approximately 5–6%) than its release from NHP407_MSN-CS-SIP-Ru (Figure 6C). Conversely, in acid pH environment higher differences were detected at each investigated time interval, with released Ru amounts approximately 10% higher for SHP407_MSN-CS-SIP-Ru compared to NHP407_MSN-CS-SIP-Ru.

#### *Ex vivo* Evaluation of Hybrid Hydrogel Injectability and Gelation

**FIGURE 7:**
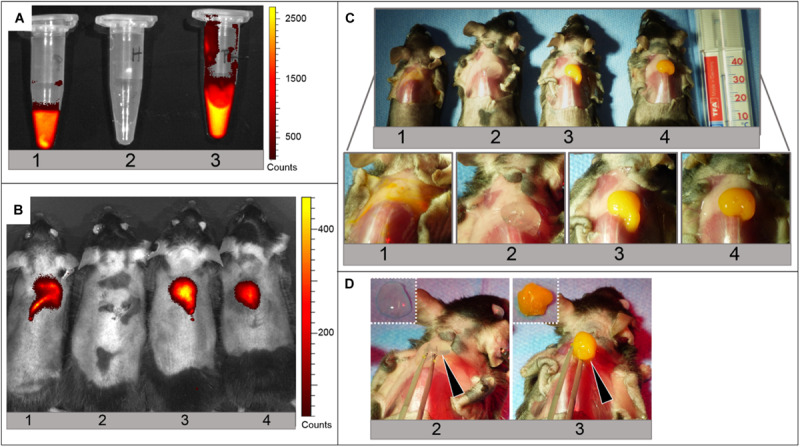
Injection and gelation of MSN-CS-SIP-Ru aqueous dispersion, SHP407 hydrogel and SHP407_MSN-CS-SIP-Ru hybrid hydrogel *in situ* into murine cadavers. **(A)** IVIS read at ex/em filter: 465 nm/Cy5.5 of MSN-CS-SIP-Ru aqueous dispersion (1), SHP407 hydrogel (2) and SHP407_MSN-CS-SIP-Ru hybrid hydrogel (3) before injection in the animal model. **(B)** IVIS read at ex/em filter: 465 nm/Cy5.5 of murine cadavers with maintained physiological body temperature subjected to injection of MSN-CS-SIP-Ru aqueous dispersion **(1)**, SHP407 hydrogel **(2)** and SHP407_MSN-CS-SIP-Ru hybrid hydrogel **(3,4)**. Only those animals injected with samples containing Ru-loaded MSN-CS-SIP exhibited a fluorescence signal. **(C)** Photographic images of the animals previously measured in the IVIS (same numbering) including a magnification of the injection site. Solid materials can be seen in animal **(2–4)**, whereas MSN-CS-SIP-Ru were broadly dispersed in the animal injected with particle suspension (animal 1). **(D)** Test of gelation of SHP407 (animal 2) and SHP407_MSN-CS-SIP-Ru (animal 3) by lifting the material up (black arrows). The solid form was maintained after the harvest (dashed pictures, top left of each image).

*Ex vivo* evaluation of hybrid hydrogel injectability, gelation and distribution was conducted according to the protocol previously adopted for SHP407 hydrogel as such. In detail, hybrid SHP407 hydrogel embedding MSN-CS-SIP-Ru was compared with SHP407 hydrogels as such and MSN-CS-SIP-Ru aqueous dispersion. Moreover, the fluorescent nature of particles’ cargo was utilized to study the detectability of the materials under the skin via an *in vivo* imaging system (IVIS). Prior to s.c. injection into murine cadavers, the fluorescence-based detectability of Ru-loaded MSN-CS-SIP within aqueous dispersion or upon incorporation within SHP407 hydrogel was confirmed using IVIS imaging (Figure 7A, ex/em filter: 465 nm/Cy5.5). Then, the just finalized mice were injected with 200 μl of material at similar concentrations, maintained at physiological body temperature and imaged after 5 min of incubation using the IVIS. Figure 7B shows the fluorescence signal obtained from Ru-loaded MSNs (ex/em filter: 465 nm/Cy5.5), which was detectable for animals 1, 3, and 4 in accordance to the nature of the injected material. In fact, animal 1 received an injection of MSN-CS-SIP-Ru dispersion, animal 2 an injection of SHP407 hydrogel as such and animals 3 and 4 were injected with the composite hydrogel SHP407_MSN-CS-SIP-Ru. By comparing animals 1 to 3 and 4, the animal that received MSN-CS-SIP-Ru suspended in physiological fluid already showed a less round shape of the fluorescence-positive injection area 5 min after injection. After the imaging, the injection site was uncovered (Figure 7C). The photographic pictures confirmed the previously seen low dispersion and good solidification of SHP407 hydrogel (animal 2), which was found to be similar to the distribution and gelation properties of the composite hydrogel (animals 3 and 4). On the contrary, MSN-CS-SIP-Ru injected as suspension in physiological fluid dispersed broadly as shown in the magnified image of the injection area. Figure 7D underlines the successful gelation of SHP407 as such and in composite with MSN-CS-SIP-Ru, as both could be lifted up as a whole using forceps (black arrows) and remained in their solid form after harvest from the injection site (Figure 7D, dashed pictures, top left).

## Discussion

In a scenario characterized by the progressive development and optimization of patient-specific smart therapeutic approaches, there is an urgent need of *ad hoc* designed injectable formulations with the potential to be finely tuned according to the pathological environment. Additionally, these injectable therapeutics could be designed to exhibit sensitivity to different physico-chemical stimuli, thus making them able to properly adapt their properties in response to external environment characteristics, with a consequent capability to smartly exert their function in the target tissue/organ. Within this complex and challenging scenario, in this work we attempted to design and thoroughly characterize a new injectable formulation responding to the aforementioned demands by exploiting the custom-made nature of its components. In fact, we combined amphiphilic PEU-based thermo-sensitive sol-gel systems with MSNs coated with a SIP providing them sensitivity to acid pH environments. Custom-made PEUs were used as alternative to commercially available Poloxamers^®^ to develop thermo-sensitive hydrogels with improved gelation properties (i.e., lower critical gelation concentration, faster gelation in physiological conditions), mechanical strength and residence time in aqueous environments (Boffito et al., 2016). Additionally, PEU versatile chemistry was exploited to introduce free amines along the polymer backbone to provide the resulting material with an enhanced sensitivity to external pH environment. On the other hand, MSNs have been selected for their easy synthesis route, tunable pore morphology and dimension, suitability to surface functionalization to expose specific functional moieties and high surface area and pore volume, which are responsible for their high loading capacities of different payloads (e.g., drugs, growth factors) (Narayan et al., 2018; Zhou et al., 2018; Xu et al., 2019; Manzano and Vallet-Regí, 2020). However, mesoporous particles exhibit an open porous network that usually makes payload diffusion out from them very fast. In this regard, the introduction of gatekeepers closing the pore entrance and opening on-demand in the presence of specific conditions has been reported as a valuable strategy to control payload release (Wen et al., 2017). In this work, MSNs were surface functionalized with a self-immolative polymer caging their pore mouth and undergoing head to tail degradation in the presence of acid pH, thus inducing pore opening and acid-pH triggered cargo release, as already demonstrated by Gisbert-Garzarán et al. (2017). According to a bottom up approach, the key components of the therapeutic formulation, i.e. the hydrogel and the particles, were first designed and characterized as single entities and then they were assembled to develop hybrid sol-gel systems answering to the previously described needs.

An amphiphilic water soluble PEU with acronym NHP407 was synthesized using Poloxamer^®^ 407, HDI and N-Boc serinol as building blocks. Successful NHP407 synthesis was proved by ATR-FTIR spectroscopy, SEC analyses and ^1^H NMR spectroscopy. The comparison between native P407 and NHP407 ATR-FTIR spectra proved the synthesis of a PEU containing P407 blocks. Indeed, in addition to P407 typical absorption peaks, new bands characteristic of urethane bonds appeared in NHP407 spectrum (Supplementary Figure S1). Additionally, complete consumption of isocyanate groups was proved by the absence of –N=C=O characteristic peak around 2200–2300 cm**^–^**^1^. Similarly, ^1^H NMR spectrum of NHP407 (Supplementary Figure S2) showed the characteristic peaks of all the building blocks used for its synthesis, i.e., resonances ascribable to the PEO and PPO segments of P407 and to the methylene protons of HDI blocks and serinol moieties (Caddeo et al., 2019). In addition, methyl protons of Boc caging groups showed their typical resonance at 1.37 ppm, in the form of a sharp singlet overlapped to HDI signals (Caddeo et al., 2019). Finally, the successful binding of NHP407 building blocks through urethane bonds was demonstrated by the signal within the spectral range between 5.65 and 5.73, which is typical of N-H groups of urethane groups (Qin et al., 2019). However, also side-reactions leading to urea bond formation occurred during NHP407 synthesis, as proved by the presence of a signal in its ^1^H NMR spectrum within the spectral region from 7.00 to 7.20 ppm (attributed to urea N-H groups) and the peak at 1630 cm**^–^**^1^ in its ATR-FTIR spectrum (ascribed to urea carbonyl groups) (Supplementary Figures S1, S2; Laurano et al., 2020). Nevertheless, despite urea by-product formation, a high molecular weight polymer with a narrow molecular weight distribution was obtained (NHP407 ${\bar{\text{M}}}_{\text{n}}$ and D were measured to be 44600 Da and 1.42, respectively). NHP407 was designed to expose Boc-protected amines along its polymer chains, which could be made available through a deprotection reaction in acid conditions, similarly to protocols habitually adopted during peptide synthesis. In this work, the protocol for Boc caging group removal from NHP407 chains was optimized to maximize free amine exposure, while avoiding detrimental polymer degradation (see Supplementary Material). In the optimized conditions, NHP407 was treated in a CHCl_3_/TFA mixture at 90/10 v/v, obtaining SHP407. ATR-FTIR spectrum of SHP407 was completely overlapped to that of native NHP407, confirming the retention of PEU chemical structure and the complete removal of both CHCl_3_ and TFA (Supplementary Figure S1). Similarly, also ^1^H NMR spectrum of SHP407 sample was completely overlapped to that of native NHP407, with the exception of the peak attributed to the methyl protons of Boc caging groups at 1.37 ppm (Supplementary Figure S2). This peak, indeed, significantly decreased its intensity in SHP407 compared to NHP407, thus proving an almost complete removal of the Boc groups (deprotection yield > 90%). SEC analyses evidenced a slight decrease in number average molecular weight upon the deprotection reaction, which, however, fell within the typical SEC analysis error (approximately 10%) (Trathnigg, 2000). The number of exposed free amines along SHP407 chains turned out to be 3.07E18 ± 1.63E17 -NH_2_/g_SHP407_ corresponding to the 93–94% of the total theoretical number of amines present along PEU chains (i.e., approximately 2–3 primary amines exposed per each SHP407 chain), in agreement with ^1^H NMR analyses. SHP407 and NHP407 were then used to prepare thermo-sensitive sol-gel systems at 15% w/v polymer concentration in a physiological solution/PBS/buffer at pH 8 mixture at 80/10/10 volume ratio. This polymer concentration was selected based on the previous characterization performed on NHP407-based hydrogels by Boffito et al. (2016), which evidenced the high potential of this formulation as injectable fast-gelling hydrogel. On the other hand, the composition of the aqueous medium used to solubilize the polymers was optimized to force hydrogel initial pH toward a neutral value (thus protecting the SIP from degradation), while maximizing the possibility to change its pH in response to environmental pH value being a not-buffered solution. Qualitative characterization through tube inverting test highlighted that SHP407-based formulation showed a slightly increased Lower Critical Gelation Temperature and gelation time at 37°C compared to NHP407-based system. These results were further confirmed by frequency sweep tests conducted at 25, 30, and 37°C (Figure 1C and Table 1) which evidenced a slightly slowed down kinetics of SHP407-based gel formation compared to NHP407-based one (e.g., at 25°C ω_G__′__/G__″__crossover_ of SHP407 and NHP407 sol-gel systems was measured to be 32.25 and 27.46, respectively). Furthermore, also the temperature of gelation onset (T_onset_) was slightly higher in SHP407 sol-gel system compared to NHP407 one (T_onset_ of 16.03 and 16.36), respectively (Figure 1B and Table 2). This different behavior can be probably correlated to the different hydrophobicity of NHP407 and SHP407: being NHP407 more hydrophobic than SHP407 due to the presence of Boc caging groups, the conditions required to induce polymer chains rearrangement into micelles were more easily reached in NHP407 aqueous solution compared to SHP407 one. Conversely, SHP407-based micelles required slightly more time and higher temperature to form, but then they turned out to be able to easily and quickly arrange into a gel network similarly to NHP407 micelles. In fact, at 37°C both NHP407 and SHP407 systems exhibited ω_G__′__/G__″__crossover_ values lower than 0.1 rad/s and turned out to be in the gel state, although a not complete gel development was achieved. This result can be correlated to the presence in SHP407-based system of a higher number of hydrogen bonds due to the exposure of free -NH_2_ groups, which contribute together with hydrophobic interactions to the progressive chain arrangement into micelles and their consequent aggregation to finally form a gel (Aoki et al., 1994; Laurano et al., 2019). This high degree of physical crosslinking within SHP407 gel was probably also responsible for its lower resistance to applied deformation (γ_L_ of 18.6 and 11.6% for NHP407 and SHP407 gels, respectively) and Yield Stress (YS of 1790 and 875 Pa for NHP407 and SHP407 gels, respectively) compared to NHP407-based one (Figure 1A). Despite these slight differences, the G′, G″ and viscosity trends reported in Figure 1 and the data summarized in Tables 1, 2 clearly evidenced that the exposure of free amines along PEU chains did not detrimentally affect the thermo-responsiveness of SHP407 hydrogel that retained the capability to quickly undergo gelation in physiological conditions. As the final hybrid formulation has been designed to release the payload encapsulated into MSNs in response to a pH trigger, the capability of both NHP407 and SHP407 gels to transfer pH changes from the surrounding aqueous environment toward their core was quantitatively evaluated through hydrogel pH measurements upon incubation with buffered solutions at pH 5 or 7.4 for predefined time intervals (Figures 2A–C). The progressive pH gradient through gel thickness was also visually analyzed using pH indicators (Figure 2D). The exposure of free amino groups along SHP407 chains effectively accelerated the transfer of acid pH through gel thickness compared to NHP407-based system. Indeed, faster pH variation kinetics (hydrogel pH change defined according to Eq. 1 within the first 30 min incubation in pH 5 environment was measured to be 42 and 52% for NHP407 and SHP407 gels, respectively) and gradient movement toward the gel core were observed in SHP407-based hydrogel compared to NHP407-based one, probably as a consequence of the progressive protonation of exposed -NH_2_ along SHP407 chains in acid environment. However, after this initial accelerated pH change in SHP407-based gel up to approximately 45 min incubation, the trend of pH of both kinds of gels became almost the same, with the pH value progressively tending to 5. On the other hand, Boc-deprotection did not affect the behavior of the gels in contact with a neutral pH environment, with no statistically significant differences. Gel swelling and stability in aqueous media were evaluated in the same conditions used to evaluate pH transfer potential. Due to the physical interactions responsible for micelle packing and gel network formation, two distinct and concurrent phenomena can be distinguished upon gel incubation in an aqueous environment, i.e., swelling and erosion/dissolution. In fact, in a watery environment, the gel network tends to absorb fluids from the surrounding environment, undergoing swelling. However, absorbed water molecules also induce a progressive dissolution of the polymer chains, which constitute the micelles, resulting in erosion/dissolution of the gels and therefore a decrease in hydrogel dry weight. The trends of swelling and dissolution/degradation (Figures 3A,B) of NHP407 and SHP407 gels were similar with dissolution/erosion phenomena becoming predominant over swelling with increasing incubation time. No clear dependence of gel swelling/stability over the pH of the surrounding aqueous medium was observed. Indeed, irrespective of the pH of the surrounding aqueous medium, SHP407-based gels exhibited an increased destabilization compared to NHP407-based ones. This different behavior can be associated to SHP407 increased hydrophilicity and water-solubility, resulting from the removal of Boc caging groups rather than to a clear pH-responsiveness induced by the exposure of amino groups along polymer backbone. Hence, although the exposed primary amines along SHP407 chains made pH transfer through gel thickness faster, they did not provide the resulting gels with a marked pH-sensitivity, which would have conferred higher swelling to SHP407 gels compared to NHP407 systems in acid environment, as a consequence of the electrostatic repulsion forces arising among micelles upon amine protonation in acid media. Increasing the number of exposed functionalities along polymer chains would lead to the design of hydrogels with further accelerated pH change capability in acid media and significantly increased swelling potential in low pH environment. To achieve this goal different approaches could be adopted. For instance, the protocol for polymer synthesis could be optimized to introduce a higher number of amine-containing building blocks in each polymer chain. In this regard, Laurano et al. have recently reported a modified synthesis protocol resulting in the introduction along the backbone of a P407-based PEU of approximately 10E20 secondary amino groups/g_PEU_ (Laurano et al., 2020). Another possible route to be investigated consists in adapting the plasma treatment protocol developed by Laurano et al. (2019) to expose primary amines along polymer backbone using allylamine as monomer (Jeong et al., 2019) or ammonia gas (Mahmoudifard et al., 2017). In view of the final application of the developed formulation, SHP407 hydrogel injectability, dispersion and gelation *in situ* within an organism were demonstrated *ex vivo* using murine cadavers (Figure 4). Results of *ex vivo* studies concerning dispersion and gelation of SHP407 sol-gel systems were in line with *in vitro* studies, since proper solidification after 5 min of incubation in a 37°C environment was observed. The dispersion of SHP407 gel was quite low, as demonstrated by the spherical shape of the material upon visual inspection (Figure 4B). For future usage of the SHP407 as a place-keeper of e.g., pharmacologically active carrier-drug systems, the restricted dispersion is of outmost importance, since the location of the intervention would need to be precise and controllable. Moreover, penetration of deeper underlying tissues would not be favorable. Creating a femoral osteotomy and applying the SHP407 hydrogel into the fracture gap allowed for a more advanced application and confirmed the beneficial gelation and dispersion properties, as the hydrogel remained in the gap area and did not penetrate the tissues underneath the femur (Figure 4D).

As second component of the final hybrid formulation, pH-responsive nanoparticles were prepared as potential drug delivery systems by grafting a self-immolative polymer on the surface of mesoporous silica nanoparticles. The production of MSN-CS-SIP was carried out by first synthesizing and characterizing its building blocks, i.e., MSNs and SIP (see Supplementary Material). MSNs were then surface functionalized with the SIP in a two-step procedure according to Scheme 2. XRD patterns highlighted that the typical hexagonally ordered mesostructure of the particles was unaffected by the coating procedure (Supplementary Figure S3A). ATR-FTIR spectrum of MSN-CS-SIP sample exhibited the characteristic absorption bands of the SIP (i.e., CH and C=O stretching vibrations at approximately 3050 and 1630 cm**^–^**^1^, respectively), thus proving the successful grafting of the SIP on MSN surface (Supplementary Figure S3C). Further confirmation of the presence of the polymeric coating covering the surface of the nanoparticles was provided by N_2_ adsorption analysis (Supplementary Figure S3A) that evidenced a reduction of the characteristic textural parameters after the SIP coating (specific surface area of MSNs and MSN-CS-SIP were measured to be *ca*. 1000 vs. *ca*. 650 m^2^/g, respectively), and TG analyses which showed the presence of a higher amount of organic material (corresponding to an increased weight loss within the 100–600°C temperature range) in MSN-CS-SIP compared to MSNs (Supplementary Figure S3B).

NHP407- and SHP407-based hydrogels encapsulating MSN-CS-SIP were finally prepared at 15% w/v and 5 mg/ml polymer and particle concentration, respectively. First, both NHP407_MSN-CS-SIP and SHP407_MSN-CS-SIP hydrogels were rheologically characterized to evaluate the effect of particle addition on the gelation potential and kinetics of the hybrid formulations (Figure 5). Particle embedding within PEU sol-gel systems turned out to affect both resistance to applied deformation and gelation kinetics of the resulting formulations. However, no detrimental effects hindering the transition from the sol to the gel state were observed. In fact, similarly to NHP407 and SHP407 hydrogels as such, both NHP407_MSN-CS-SIP and SHP407_MSN-CS-SIP systems appeared to be in the gel state at 37°C (ω_G__′__/G__″ crossover_ lower than 0.1 rad/s). Additionally, both the formulations did not exhibit a G′ trend independent over frequency, proving that the gel network was not completely developed at physiological temperature (Figure 5C and Table 3). However, in terms of gelation kinetics, MSN-CS-SIP loading within the hydrogels turned out to have different effects on SHP407 and NHP407 sol-gel systems. SHP407 hybrid formulation showed a faster sol-to-gel transition compared to virgin SHP407 hydrogel, as suggested by the lower ω_G__′__/G__″__crossover_ values it exhibited at both 25 and 30°C (Figure 5C and Table 3). This behavior could be probably correlated to the presence of the polymeric coating covering the particles, which made them able to take part to the gelation process, acting as additional crosslinking points within the gel network thanks to the hydrogen bonds arising between them and SHP407 micelles. This hypothesis was further supported by the observed decrease in T_onset_ (from 16.36 to 15.7°C upon particle encapsulation) (Figure 5B and Table 4). Differently, particle embedding within NHP407-based sol-gel system had an opposite effect, as demonstrated by the slightly higher ω_G__′__/G__″__crossover_ values of NHP407_MSN-CS-SIP system with respect to NHP407 control hydrogel at both 25 and 30°C. The different behavior of NHP407-based hybrid hydrogel could be ascribed to the presence of Boc groups that acted as obstacles to H-bond formation due to steric hindrance and their caging activity of amino groups that, conversely, in SHP407 were available for hydrogen bonding. Irrespective of the nature of the constituent polymer, at 37°C the particles acted as defects within the gel network, lowering its resistance to applied deformation and Yield Stress (Figure 5A). These results further corroborated the previous hypothesis on the formation of hydrogen bonds between the SIP and SHP407 chains. In fact, a lower decrease of both γ_L_ and YS was observed for SHP407-based system compared to NHP407-based one (i.e., upon particle addition, γ_L_ decreased of 84.8 and 37.5% in NHP407- and SHP407-based systems, respectively. Decrease in YS was 85.4 and 49.5% in NHP407- and SHP407-based systems, respectively). Finally, the capability of hybrid formulations to release the payload previously encapsulated in MSN-CS-SIP was characterized in pH 5 and pH 7.4 aqueous media (Figure 6). Despite encapsulation within the gel phase, SIP-coated MSNs retained the capability to release their cargo. In fact, at both pH 5 and pH 7.4 environments, the Ru dye was progressively released from the hybrid hydrogels with no burst release. In accordance with the pH-responsiveness of the SIP, triggered Ru dye release in acid environment was successfully achieved from both NHP407_MSN-CS-SIP-Ru and SHP407_ MSN-CS-SIP-Ru (Figure 6A), thus proving that the progressive SIP degradation at acid pH and the opening of the pore entrances were not hindered by the embedding of the particles within a hydrogel phase. Furthermore, the exposure of free amino groups along SHP407 polymer chains turned out to effectively enhance Ru release from SHP407_MSN-CS-SIP-Ru compared to NHP407_MSN-CS-SIP-Ru (Figure 6B). This result is in agreement with the accelerated decrease in pH observed in SHP407 sol-gel systems compared to NHP407 ones, during incubation in acid pH environment (Figure 2A). On the other hand, no significant differences in Ru release were observed in neutral pH medium, in accordance with the similar pH trend SHP407 and NHP407 hydrogels exhibited upon incubation in this environment (Figure 2B). *Ex vivo* injectability and gelation were then assessed also for SHP407_MSN-CS-SIP-Ru hybrid formulation (Figures 7C,D). The capability of the hydrogel vehicle phase to localize the therapeutic formulation in the target area was demonstrated by s.c. injecting the hybrid formulation and comparing this to a MSN-CS-SIP-Ru aqueous dispersion that, in fact, tended to broadly disperse immediately upon application (Figure 7C). Additionally, the loading of the fluorescent cargo Ru into the MSNs allowed for a fluorescence-based detection of the particle dispersion as such, but also upon loading into the hydrogel, both *in vitro* (Figure 7A) as well as *in vivo* (Figure 7B). Hence, SHP407 gel showed ability to transmit fluorescent signals, which can be a crucial feature for potential proof-of-concept *in vivo* payload release studies.

## Conclusion

The optimal therapeutic drug-release formulation should recapitulate in a sole device several features and fulfill a specific plethora of strict requirements. First, it must be easy to handle, injectable and able to completely fill body cavities or defects. Second, the capability to change its state and adapt itself to different scenarios is mandatory to allow injection, good distribution in the target tissue/organ and adequate residence time to properly exert its function. Third, it must progressively release its payload in a controlled way, avoiding undesired burst release and ensuring the delivery of active therapeutic agents within the target tissue/organ at a suitable concentration within the therapeutic window for the proper time frame. Last, it should possess high versatility allowing a wide possibility of tuning its composition. As an additional feature, smartness could make therapeutic formulations able to actively respond to external stimuli, such as physical and biochemical cues, thus allowing them to change their properties, with the consequent possibility to trigger and modulate cargo release. Within this challenging and highly demanding scenario, in this work we succeeded in developing formulations meeting the aforementioned demands and showing concurrent sensitivity to temperature and environmental pH. Thermo-responsiveness was successfully provided through the use of an amphiphilic polymer as hydrogel forming material to make the resulting formulations able to quickly gel in physiological conditions. On the other hand, mesoporous silica particles coated with a properly designed self-immolative polymer provided the resulting formulation with responsiveness to acid pH environment, resulting in accelerated delivery of their payload in specific conditions. *Ex vivo* demonstrated injectability, gelation and confined distribution of the newly designed formulations represent additional key features for their application in the biomedical field. Moreover, in view of a potential *in vivo* application, the here-developed formulations and their degradation products can be hypothesized to be suitable for complete excretion from the body. Indeed, mesoporous silica nanoparticles have been demonstrated in literature to undergo degradation/dissolution in simulated biological media, with a progressive increase in pore size and decrease in both porosity and surface area (Cauda et al., 2010; Chen et al., 2015). On the other hand, the poly(ether urethane)s used as hydrogel-forming materials are susceptible to progressive hydrolytic or oxidative degradation, which could be also triggered by local environment properties [e.g., local pH (Mesa et al., 2005; Yang et al., 2006) and presence of specific ionic species (Boffito et al., 2019b)]. On the other hand, being dissolution the main phenomenon responsible for physical hydrogel disassembling in aqueous media in the short-term, also full-length polymer chain will probably circulate in the body and expected to be excreted via renal clearance [albumin -66 kDa- is usually referred to as a good estimate for glomerular molecular weight cut-off (Lin, 2009)].

The high potential of the here-proposed approach lies in its many compositional degrees-of-freedom and the custom-made nature of its constituents. The high versatility of poly(urethane) chemistry opens the way to the possibility to further increase the sensitivity of the gel phase to the surrounding environment through a proper selection of the building blocks or additional functionalization procedures. For instance, enzyme-sensitive blocks [e.g., matrix metalloproteinase-sensitive peptide sequences (Lutolf et al., 2003)] could be introduced into the polymer backbone allowing a further control over the stability of the system upon *in vivo* injection. On the other hand, according to recently reported data by some authors of the present work (Pontremoli et al., 2018; Boffito et al., 2019b), thermo-sensitive hydrogels based on custom-made poly(ether urethane)s have been demonstrated to allow mesoporous particle encapsulation at high concentration, thus making it possible to modulate cargo loading within a wide concentration range. In addition, the mesoporous nature of embedded particles allows the encapsulation of huge amounts of payload, thus further increasing the potential of the here-developed injectable formulations. Finally, also the custom-made nature of the self-immolative polymers opens the way to a further tuning of payload release profile. The multi-component nature of these hybrid hydrogels and the custom-made nature of its constituents will thus allow in the future to assemble *ad hoc* formulated therapeutics answering to the specific needs of each patient.

## Data Availability Statement

The raw data supporting the conclusions of this article will be made available by the authors, without undue reservation, to any qualified researcher.

## Ethics Statement

The animal study was approved by the local animal protection authorities (Landesamt für Gesundheit und Soziales (LaGeSo); permit number: G 0293/17) and was performed in accordance with the German Animal Welfare Act.

## Author Contributions

This is a multi-disciplinary work that has been conducted by three main institutions (Politecnico di Torino, POLITO, Universidad Complutence de Madrid, UCM, and Charité - Universitätsmedizin Berlin, CHARITÉ). GC was responsible for POLITO’s unit and coordinated the research activities, MB, AT, CT-T, and RL designed the thermo-sensitive hydrogels and executed all the experimental activities related to hydrogel fabrication and characterization. They were also responsible for the design and characterization of hybrid sol-gel systems. CC performed and interpreted ^1^H NMR analyses. MV-R and MM were responsible for UCM’s activities. MG-G synthesized and characterized pH-sensitive mesoporous silica nanoparticles and loaded them with the fluorescent cargo. GD, KS-B, and JB were responsible for the research activities carried out by CHARITÉ units. JB and KS-B planned and executed *ex vivo* injectability and gelation tests in rodents. Data interpretation responsibility was collectively shared by all the authors. MB wrote the whole manuscript with contribution on particle synthesis and characterization and *ex vivo* tests from MG-G and JB, respectively. All authors provided critical feedback and helped shape the research, analysis and manuscript.

## Conflict of Interest

The authors declare that the research was conducted in the absence of any commercial or financial relationships that could be construed as a potential conflict of interest.

## Footnotes

1 http://www.graphpad.com
