# Supplementary material for: Hybrid Injectable Sol-Gel Systems Based on Thermo-Sensitive Polyurethane Hydrogels Carrying pH-Sensitive Mesoporous Silica Nanoparticles for the Controlled and Triggered Release of Therapeutic Agents
Source: Front Bioeng Biotechnol. 2020 May 19;8:384. doi: 10.3389/fbioe.2020.00384 (PMC7248334; doi:10.3389/fbioe.2020.00384)
Supplement: Supplementary file 1 [file Table_1.docx]

Supplementary Material

# Characterization of NHP407 and SHP407


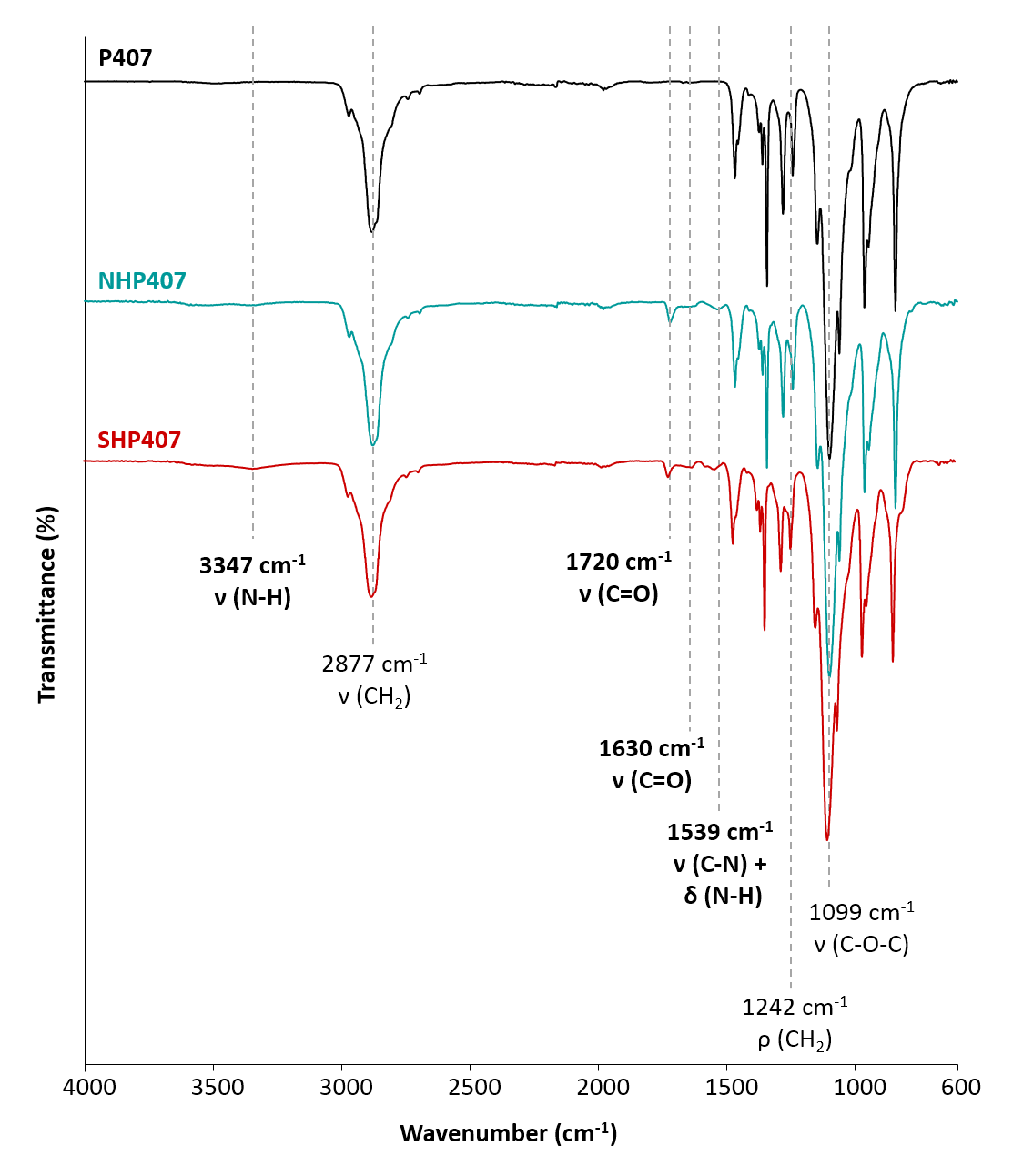


**Supplementary Figure S1.** ATR-FTIR spectra of P407 (black), NHP407 (green) and SHP407 (red). Characteristic absorption bands of urethane bonds are highlighted in bold.


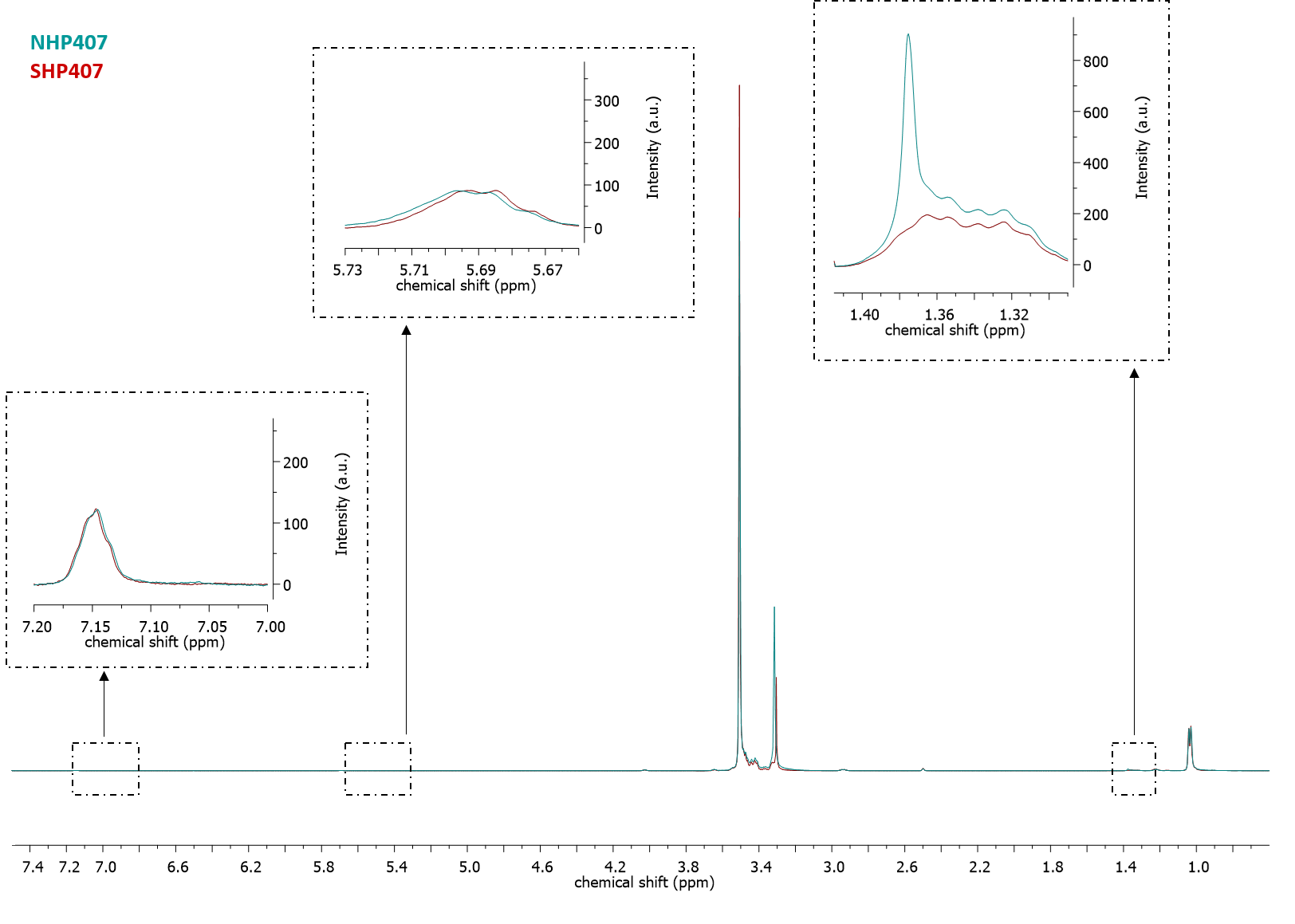


**Supplementary Figure S2.** ^1^H NMR spectra of NHP407 (green) and SHP407 (red). Magnified inserts highlight the characteristic bands of N-H groups of urethane and urea bonds, and Boc methyl groups.

# Characterization of SIP-coated mesoporous silica nanoparticles


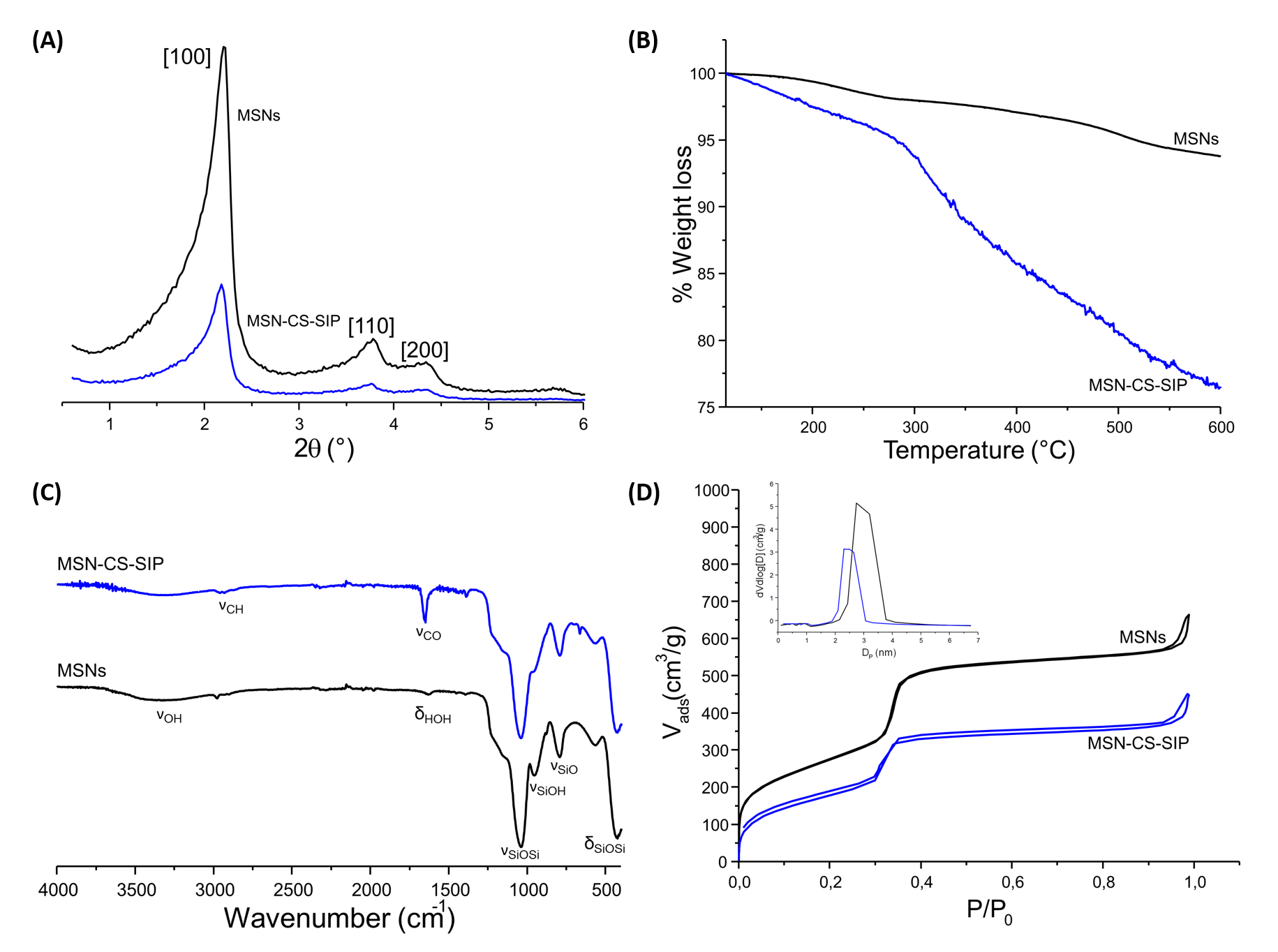


**Supplementary Figure S3.** Characterization of MSN-CS-SIP (blue) *vs*. MSNs (black). **A)** XRD. **B)** TG analysis. **C)** ATR-FTIR spectroscopy. **D)** N_2_ adsorption analysis.

# Optimization of NHP407 deprotection protocol

**Materials and Methods**

### *Exposure of free amines along poly(ether urethane) backbone*

With the aim to expose available free amines along NHP407 backbone, as-synthesized polymer was solubilized in chloroform (CHCl_3_) at room temperature (RT) for 120 minutes (under stirring at 250 rpm) and then trifluoroacetic acid (TFA) was added to the solution and the mixture was left to react for 60 minutes at RT. In order to maximize the efficiency of the deprotection treatment while minimizing the amount of TFA added, an optimization of the reaction was carried out modulating the CHCl_3_/TFA ratio. In detail, NHP407 final concentration in the CHCl_3_/TFA mixture was left constant to 4% w/v, while the CHCl_3_/TFA ratio was set at 95/5, 90/10 and 80/20 v/v. Due to the characteristic acidic behavior of CHCl_3_ (Margolin and Long, 1973)_,_ the deprotection reaction was also carried out in the absence of TFA (i.e., CHCl_3_/TFA at 100/0 v/v). At the end of the deprotection reaction, solvents were evaporated under vacuum using a rotary evaporator (Buchi Rotavapor Labortechnik AG) and the collected polymer was washed twice using chloroform (10% w/v) to completely evaporate TFA residues. Finally, the polymer was solubilized in distilled water (5% w/v) at 4 °C overnight and dialyzed (cellulose membrane cut-off 10-12 kDa, Sigma Aldrich, Italy) against distilled water for 2 days (water refresh three times/day) to completely wash out Boc groups and residual CHCl_3_ and TFA molecules. Deprotected polymer was then freeze dried (Martin Christ ALPHA 2–4 LSC) and stored under vacuum at 4 °C until use.

Hereafter, the collected polymers after the Boc-deprotection reaction will be referred to with the acronym SHP407_X%TFA where X defines the TFA percentage content present in the CHCl_3_/TFA mixture.

### *Chemical characterization*

### Attenuated Total Reflectance Fourier Transform Infrared (ATR-FTIR) spectroscopy, Size Exclusion Chromatography (SEC) and Proton Nuclear Magnetic Resonance (^1^H NMR) spectroscopy were exploited for the chemical characterization of SHP407_X%TFA samples, according to the protocols described in the paper. NHP407 was also characterized as control sample.

**Results**

With the aim to assess the integrity of PEU backbone upon treatment in CHCl_3_/TFA mixture (100/0, 95/5, 90/10, 80/20 v/v), ATR-FTIR, SEC and ^1^H NMR analyses were performed on SHP407_X%TFA samples and NHP407 as control. **Supplementary Figure S4** and **S5** report the ATR-FTIR and ^1^H NMR spectra of NHP407 (control) and SHP407_X%TFA samples, respectively.


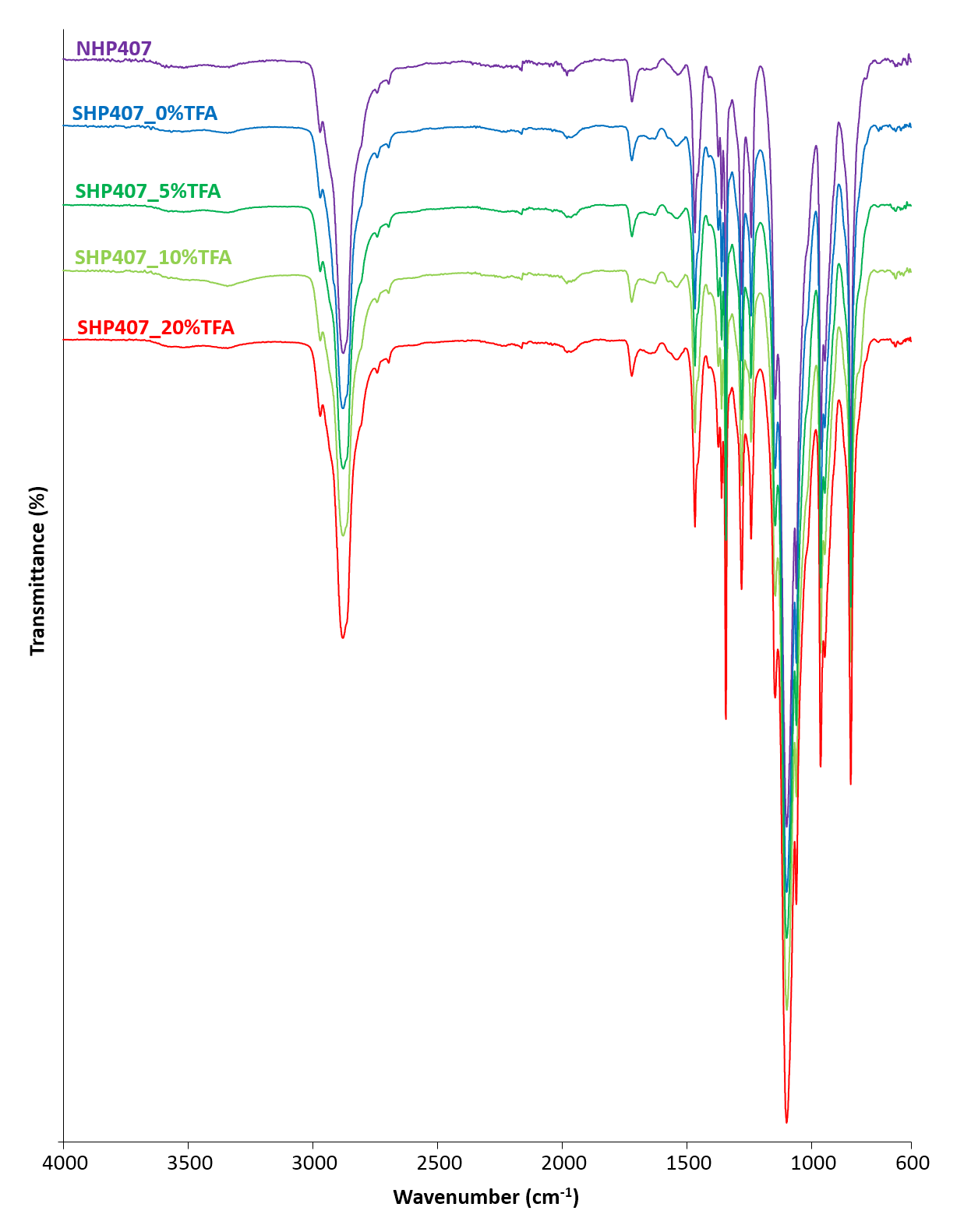


**Supplementary Figure S4.** ATR-FTIR spectra of NHP407 (control) and SHP407 PEUs (SHP407_0%TFA, SHP407_5%TFA, SHP407_10%TFA and SHP407_20%TFA) synthesized by treating NHP407 in CHCl_3_/TFA at different volumetric ratios (i.e., 100/0, 95/5, 90/10, 80/20 v/v).

ATR-FTIR spectra of SHP407_X%TFA samples were completely overlapped with that of native NHP407 and no absorption peaks ascribable to residual CHCl_3_ or TFA (e.g., C-Cl and C-F stretching vibration at 600-800 cm^-1^ and 1000-1400 cm^-1^, respectively) were detected, thus confirming the retention of PEU chemical structure and the complete removal of both CHCl_3_ and TFA.


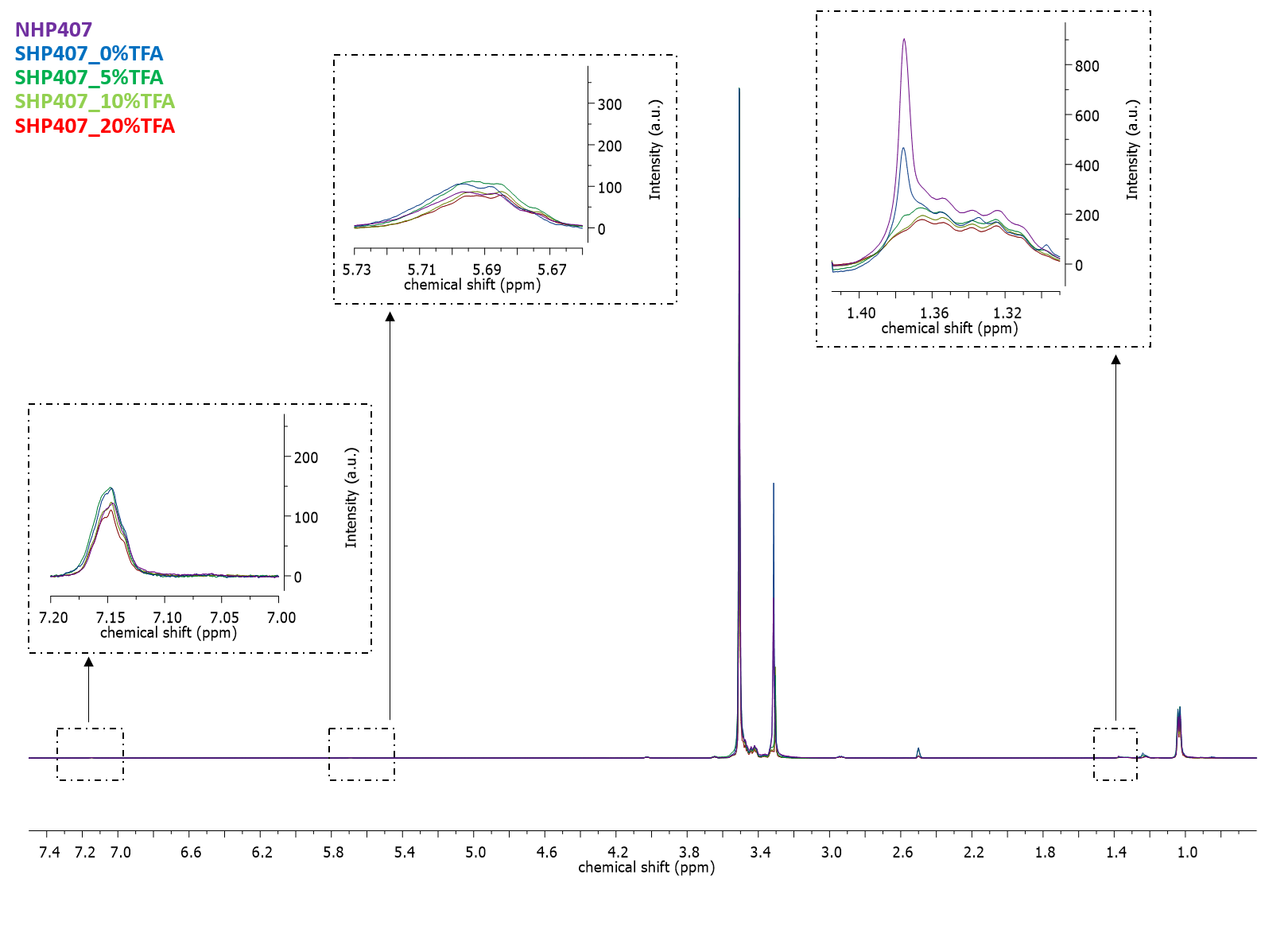


**Supplementary Figure S5.** ^1^H NMR spectra of NHP407 (control) and SHP407 poly(ether urethane)s (SHP407_0%TFA, SHP407_5%TFA, SHP407_10%TFA and SHP407_20%TFA) synthesized by treating NHP407 in CHCl_3_/TFA at 100/0, 95/5, 90/10, 80/20 v/v. Magnified inserts highlight the characteristic bands of N-H groups of urethane and urea bonds, and Boc methyl groups.

Similarly, also ^1^H NMR spectra of SHP407_X%TFA samples were completely overlapped to that of native NHP407, with the exception of the peak attributed to the methyl protons of Boc caging groups at 1.37 ppm. Indeed, upon treatment in acid conditions, the singlet at 1.37 ppm due to Boc protons progressively decreased with increasing TFA content, indicating that the reaction conditions allowed Boc removal. Irrespective of the CHCl_3_/TFA volumetric ratio adopted for Boc cleavage, all other signals in the ^1^H NMR spectra remained unaltered, proving that Boc cleavage treatment did not significantly affect polymer chemical structure. Due to its acid nature, chloroform as such (SHP407_0%TFA) turned out to be able to partially deprotect Boc-protected amines of NHP407, with a deprotection yield of approx. 50 % (evaluated as percentage decrease in the intensity of Boc group peak). At 5% v/v TFA concentration (SHP407_5%TFA), the signal ascribed to Boc groups almost completely disappeared suggesting that the addition of TFA al low concentration can effectively cleave Boc groups from NHP407, with a yield of approx. 80 %. A further decrease in the intensity of Boc proton resonance at 1.37 ppm was observed with increasing TFA concentration during the deprotection reaction to 10% v/v. No further changes in the ^1^H NMR spectrum within the spectral region between 1.31 and 1.41 ppm were observed with increasing TFA content to 20% v/v, indicating that a CHCl_3_/TFA volumetric ratio of 90/10 was sufficient to reach an almost complete Boc deprotection (deprotection yield > 90%). SEC analyses evidenced a slight decrease in SHP407_X%TFA number average molecular weight ($\bar{M}_{n}$) (from 44600 Da to approx. 41000 Da) associated with an increase in polydispersity index (D) values (from 1.42 to around 1.56) (**Supplementary Figure S6**). Although the change in $\bar{M}_{n}$ was not significant, being the typical SEC analysis error in the order of 10% (Trathnigg, 2000), the concurrent decrease in the estimated molecular weight and increase in D suggested that the deprotection treatment slightly affected the PEU integrity inducing the cleavage of its polymer chains. Additionally, this effect was dependent over TFA concentration within the CHCl_3_/TFA mixture, with $\bar{M}_{n}$ and D trends decreasing and increasing, respectively, with increasing TFA concentration and can be correlated to hydrolysis phenomena of ethylene oxide blocks of P407 occurring in strong acid conditions (Mesa et al., 2005; Yang et al., 2006).


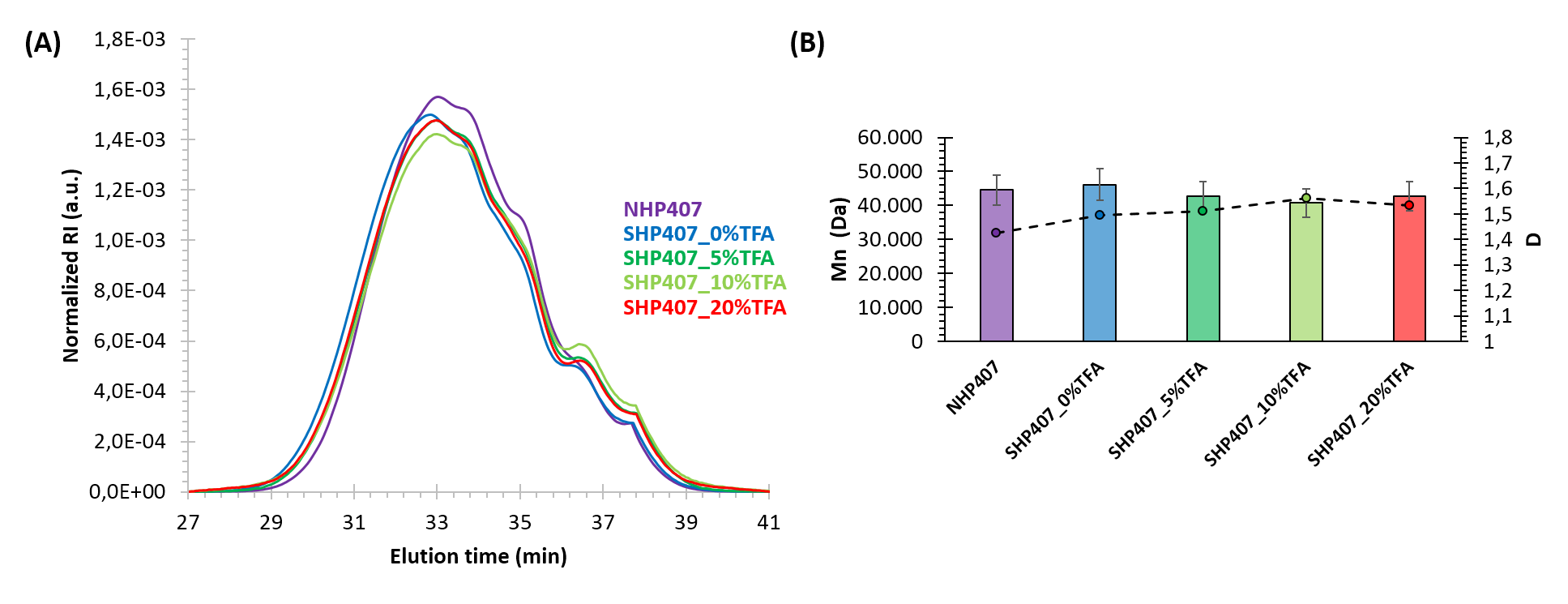


**Supplementary Figure S6.** Results of SEC analyses performed on NHP407 (control) and SHP407 poly(ether urethane)s (SHP407_0%TFA, SHP407_5%TFA, SHP407_10%TFA and SHP407_20%TFA) synthesized by treating NHP407 in CHCl_3_/TFA mixture at 100/0, 95/5, 90/10, 80/20 v/v. **(A)** Trend of normalized refractive index (RI) as a function of elution time; **(B)** Number average molecular weight ($\bar{M}_{n}$, bar chart) and polydispersity index (D, dispersion graph) values. $\bar{M}_{n}$error has been set at ±10% according to (Trathnigg, 2000).

Combining SEC and ^1^H NMR results, the CHCl_3_/TFA mixture at 90/10 volume ratio turned out to be the best compromise to maximize Boc-caging group deprotection, while minimizing polymer degradation.

In the recently published work by Laurano et al. the same deprotection conditions were employed to deprotect a similar PEU containing secondary amines along its polymer chains (Laurano et al., 2020). However, in that work only 80% deprotection yield was achieved probably because of the higher amount of amino groups present in each polymer chain (the authors adopted a modified synthesis protocol to maximize the yield of the chain extension reaction). This result suggests the need to optimize the deprotection reaction depending on the number of Boc groups to be removed (by increasing TFA content with increasing the number of amino groups to be deprotected, while saving the polymer from degradation). On the other hand, compared to the protocol published by Caddeo et al. (Caddeo et al., 2019) for a PEO-based PEU, in the present work we succeeded in achieving the same degree of deprotection using a significantly lower amount of TFA; however, not in depth comparison can be made as in that work neither SEC analyses nor exposed amino groups quantification were performed.

# Characterization of SIP, MSNs and MSN-CS

**Materials and Methods**

# *Physico-chemical characterization of SIP, MSNs and MSN-CS*

The successful synthesis of compounds **1**, **2** and **3** was verified by ^1^H NMR spectroscopy using a Bruker AV250 MHz in anhydrous deuterated dimethyl sulfoxide (DMSO-d6, 99.8% D, Sigma Aldrich). The spectra resulted from 16 scans, with 10 s relaxation time.

MSNs and MSN-CS samples were characterized by Power X-Ray Diffraction (XRD) analyses, ATR-FTIR spectroscopy, Nitrogen adsorption and desorption isotherms recording, and Thermogravimetric (TG) analyses. All characterizations were performed according to the protocols described in the paper.

MSNs were also characterized by Dynamic Light Scattering (DLS) analyses, Scanning Electron Microscopy (SEM) and Transmission Electron Microscopy (TEM) tests. In detail, particle size distribution was evaluated by analyzing a particle dispersion (1 mg/ml, 5 min sonication before analysis) with a Zetasizer Nano ZS (Malvern Instrument Ltd.) Dynamic Light Scattering (DLS) instrument. Morphological characterization was performed by Scanning Electron Microscopy (SEM) and Transmission Electron Microscopy (TEM) analyses recorded on JEOL JSM 6335F and JEOL JEM 2100 instruments, respectively.

**Results and Discussion**

### *Characterization of as-synthesized self-immolative polymer*

ABA was employed as building block for SIP synthesis owing to its well-known self-immolative properties (Toki et al., 2002; Müller et al., 2010). The synthesis of SIP involved the polymerization of a self-immolative monomer in the presence of a tin catalyst. Then, the polymer chain was endowed with pH-responsiveness using a triggering monomer as end-cap for the polymer chain. Compound **1** was obtained by reacting ABA with phenyl chloroformate. In parallel, compound **2** resulted from the reaction of ABA with BOC_2_O, yielding a monomer with the trigger already incorporated. Finally, the synthesis of the pH-responsive self-immolative polymer was mediated by DBTDL using compound **1** as monomer and compound **2** as trigger. The successful synthesis of the pH-responsive self-immolative polymer (compound **3**) and of its starting reagents (i.e., compound **1** and compound **2**) was verified by the appearance of the expected signals in ^1^H NMR spectra, which also proved the absence of undesired by-products and residual unreacted reagents (**Supplementary Figure S7**). The molecular weight of compound **3** (i.e., the SIP) was calculated from ^1^H NMR spectrum. The number of monomers per polymer chain was calculated through the ratio “benzylic hydrogens at the molecule tail *vs* those in the polymeric chain”. An average synthesis led to SIP molecules with *ca.* 20 units (*ca.* 3300 g/mol).


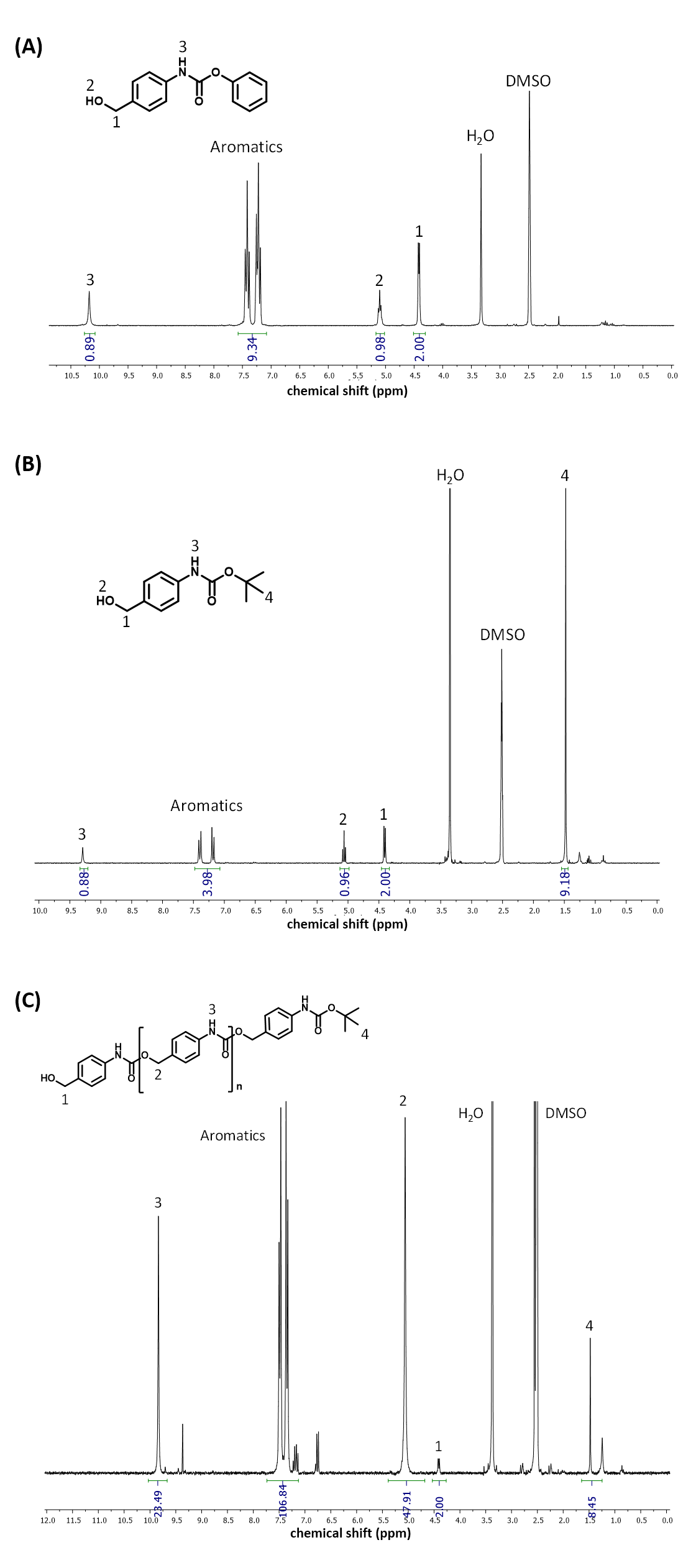


**Supplementary Figure S7.** ^1^H NMR spectra of: **A)** compound **1**. δ 10.20 (s, 1H), 7.25-7,45 (m, 9H), 5.12 (t, 1H), 4.45 (d, 2H); **B)** compound **2**. δ 9,29 (s, 1H), 7.17-7,41 (m, 4H), 5.05 (t, 1H), 4.40 (d, 2H), 1,48 (s, 9H); **C)** compound **3**. δ 9.82 (s, 10H), 7.56-7.15 (m, 49H), 5.15(s, 23H), 4.43 (d, 2H), 1.48 (s, 1H). In compound **3** ^1^H NMR spectrum the ratio between integrals of peaks 2 and 1 provided the number of monomers in the chain. Signal 4 integral provided the amount of trigger incorporated.

### *Characterization of MSNs and MSN-CSs*

SEM micrographs of MSNs demonstrated the successful synthesis of particles with homogenous shape and a size of *ca*. 200 nm (**Supplementary Figure S8A**), meanwhile the achievement of a mesoporous framework was demonstrated by TEM (**Supplementary Figure S8B**). DLS analyses revealed the absence of aggregates within MSN dispersion, and an average size of about 200 nm (polydispersity index of 0.47), in agreement with SEM micrographs.


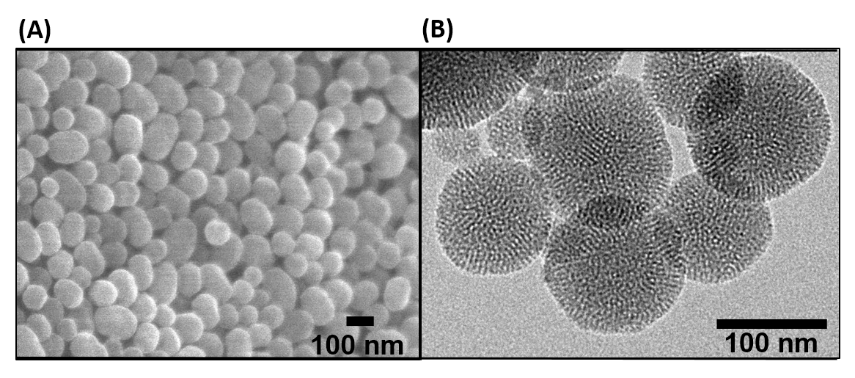


**Supplementary Figure S8.** SEM **(A)** and TEM **(B)** micrographs of MSNs.

The symmetry of the obtained porous structure was demonstrated by XRD analysis that evidenced (**Supplementary Figure S9A**) the typical diffraction peaks of the hexagonally ordered pore distribution with *p6mm* spatial group of MCM-41-like materials (Manzano et al., 2008; Vallet-Regí, 2010). Such diffraction maxima were still observable in MSN-CSs. ATR-FTIR spectrum (**Supplementary Figure S9B**) reported the typical absorption peaks of both Si-O-Si and Si-OH, which confirmed the formation of the silica backbone and the presence of silanol groups on the surface of the particles, respectively. Successful grafting of the chloro alkoxysilane on MSN surface was proved by the appearance of an additional absorption peak at 2900 cm^-1^, which can be ascribed to C-H stretching vibration. A further confirmation of successful functionalization was obtained from TG analyses, which evidenced an increment of *ca*. 9% in the weight loss of MSN-CS sample compared to MSNs as such (**Supplementary Figure S9C)**. N_2_ adsorption analysis revealed the characteristic textural properties expected for this type of material and the presence of cylindrical mesopores (Type-IV isotherm) (**Supplementary Figure S9D**) (Kruk and Jaroniec, 2001). From a morphological point of view, MSNs were characterized by a surface area of *ca*. 1000 m^2^/g and pore width and volume of 2.74 nm and *ca*. 1 cm^3^/g, respectively. A slight reduction of the textural parameters after CS grafting was observed, with pore width and surface area decreasing to approx. 2.66 nm and 915 m^2^/g, respectively.


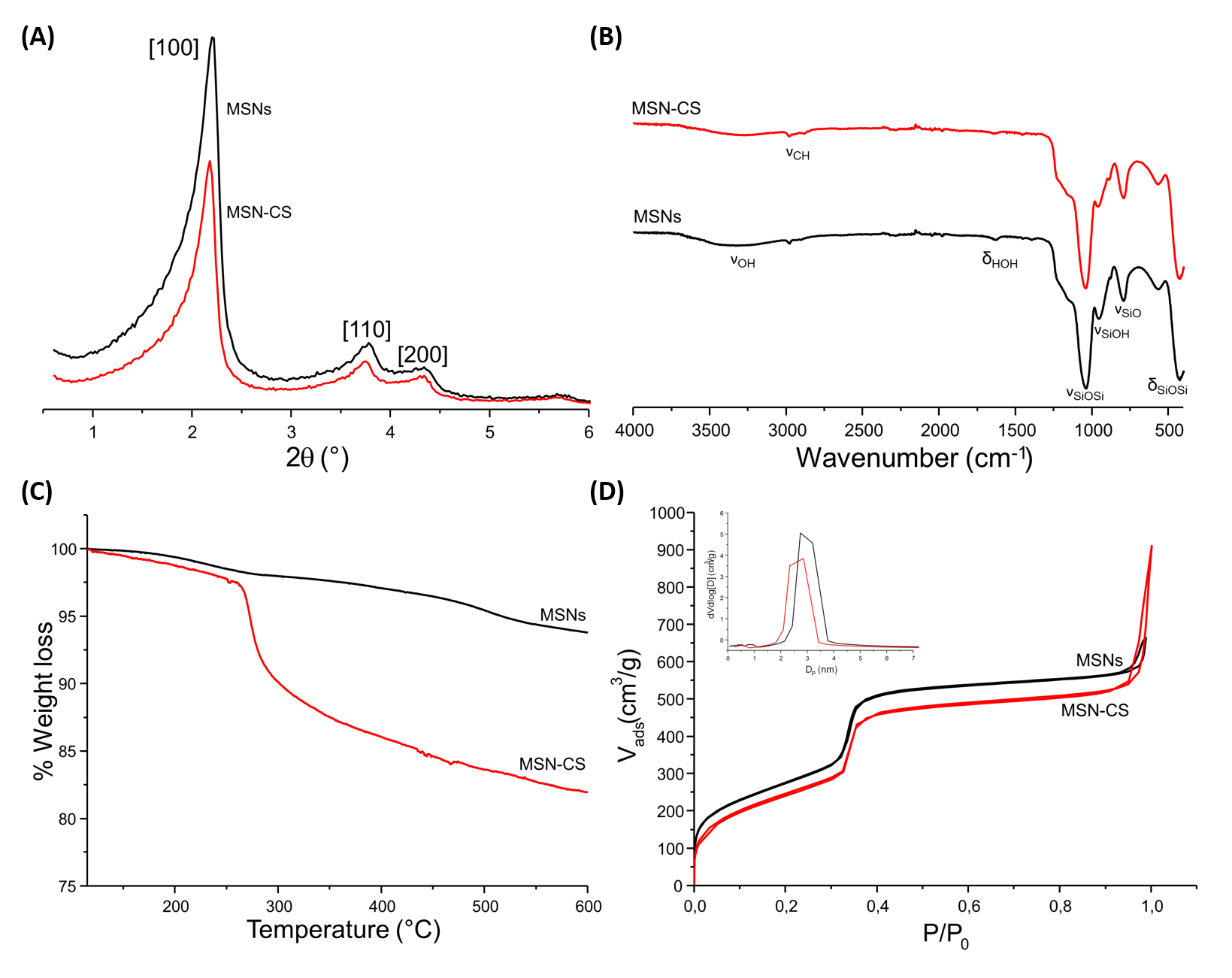


**Supplementary Figure S9.** Characterization of MSNs *vs.* MSN-CS. **A)** XRD. **B)** ATR-FTIR spectroscopy. **C)** TG analysis. **D)** N_2_ adsorption analysis.

# References

Caddeo, S., Mattioli-Belmonte, M., Cassino, C., Barbani, N., Dicarlo, M., Gentile, P., et al. (2019). Newly-designed collagen/polyurethane bioartificial blend as coating on bioactive glass-ceramics for bone tissue engineering applications. *Mater. Sci. Eng. C* 96, 218-233. doi:10.1016/j.msec.2018.11.012.

Kruk, M., and Jaroniec, M. (2001). Gas adsorption characterization of ordered organic-inorganic nanocomposite materials. *Chem. Mater.* 13, 3169–3183. doi: 10.1021/cm0101069.H

Laurano, R., Cassino, C., Ciardelli, G., Chiono, V., and Boffito, M. (2020). Polyurethane-based thiomers: A new multifunctional copolymer platform for biomedical applications. *React. Funct. Polym.* 146, 104413. doi:10.1016/j.reactfunctpolym.2019.104413.

Manzano, M., Aina, V., Areán, C. O., Balas, F., Cauda, V., Colilla, M., et al. (2008). Studies on MCM-41 mesoporous silica for drug delivery: Effect of particle morphology and amine functionalization. *Chem. Eng. J.* 137, 30–37. doi: 10.1016/j.cej.2007.07.078.

Margolin, Z., and Long, F. A. (1973). Acidic behavior of chloroform. *J. Am. Chem. Soc.* 95, 2757-2762. doi:10.1021/ja00790a001.

Mesa, M., Sierra, L., Patarin, J., and Guth, J. L. (2005). Morphology and porosity characteristics control of SBA-16 mesoporous silica. Effect of the triblock surfactant Pluronic F127 degradation during the synthesis. *Solid State Sci.* 7, 990-997. doi:10.1016/j.solidstatesciences.2005.04.006.

Müller, I. A., Kratz, F., Jung, M., and Warnecke, A. (2010). Schiff bases derived from p-aminobenzyl alcohol as trigger groups for pH-dependent prodrug activation. 51, 4371–4374. doi: 10.1016/j.tetlet.2010.06.055.

Toki, B. E., Cerveny, C. G., Wahl, A. F., and Senter, P. D. (2002). Protease-mediated fragmentation of p-amidobenzyl ethers: A new strategy for the activation of anticancer prodrugs. *J. Org. Chem.* 67, 1866–1872. doi: 10.1021/jo016187+.

Trathnigg, B. (2000). "Size-exclusion chromatography of polymers", in *Encyclopedia of Analytical Chemistry*, ed Meyers R. A. (Chichester, Jonh Wiley & Sons Ltd), 8008–8034.

Yang, B., Guo, C., Chen, S., Junhe, M., Wang, J., Liang, X., et al. (2006). Effect of acid on the aggregation of poly(ethylene oxide)-poly(propylene oxide)-poly(ethylene oxide) block copolymers. *J. Phys. Chem. B* 110, 23068-23074. doi:10.1021/jp0634149.

Vallet-Regí, M. (2010). Nanostructured mesoporous silica matrices in nanomedicine. *J. Intern. Med.* 267, 22–43. doi: 10.1111/j.1365-2796.2009.02190.x.
